# Peripheral nerve injury-induced remodeling of the tumor-associated macrophages promotes immune evasion in breast cancer

**DOI:** 10.1186/s13046-025-03545-x

**Published:** 2025-10-06

**Authors:** Yongxue Jiang, Wenfeng Zeng, Yaxin Feng, Xiaoting Deng, Jiayi Wang, Haiyu Liu, Boying Gao, Dexi Bi, Zifei Liu, Chaoqun Yang, Minxia Chen, Tang Li, Houying Chen, Yuxi Zhang, Luyuan Liang, Jiannan Xu, Wen Deng, Zeyu Yao, Wei Wu, Liyan Lao, Jianing Chen, Penghan Huang

**Affiliations:** 1 Guangdong Provincial Key Laboratory of Malignant Tumor Epigenetics and Gene Regulation, Medical Research Center, Sun Yat-Sen Memorial Hospital, Sun Yat-Sen University, Guangzhou, 510120 China; 2 Breast Tumor Center, Sun Yat-Sen Memorial Hospital, Sun Yat-Sen University, Guangzhou, 510120 China; 3 Zenith Institute of Medical Sciences, Guangzhou, 510120 China; 4 Harbin Medical University, Harbin, Heilongjiang 150081 China; 5 School of Basic Medical Sciences, Southern Medical University, Guangzhou, 510515 China; 6 Department of Dermatology, Zhejiang Provincial Dermatology Hospital, Huzhou, 313000 China; 7 Center for Biotherapy, Sun Yat-sen Memorial Hospital, Sun Yat-sen University, Guangzhou, China; 8 Department of Breast Surgery of the Sixth Affiliated Hospital, South China University of Technology, Guangzhou, China

**Keywords:** NFL, A nerve damage biomarker, Reprograms TAMs to induce CD8^+^T cell senescence, Revealing a novel neuro-immune axis in breast cancer

## Abstract

**Background:**

Peripheral nerve damage is intricately linked to the progression of various solid tumors. However, its effect on antitumor immunity and precise underlying mechanisms remain poorly understood. This study aimed to elucidate the effect of peripheral nerve damage and its subsequent immune-modulating effects influence on breast cancer progression.

**Methods:**

We analyzed nerve injury markers in the TCGA-BRCA database and clinical samples. In vivo experiments were conducted using orthotopic breast cancer models with chemical sympathetic denervation (6-OHDA) or nerve lysate/neurofilament light chain (NFL) treatment, where NFL was identified as a key effector molecule through mass spectrometry screening. The tumor microenvironment was evaluated by flow cytometry, multiplex immunohistochemistry, and single-cell RNA sequencing. In vitro co-culture systems were established to investigate the effects of NFL on macrophages and CD8^+^ T cells, with transcriptomic profiling revealing that NFL-activated macrophage supernatants induced CD8^+^ T cell senescence via NF-κB signal pathway activation.

**Results:**

Peripheral nerve injury was associated with poor prognosis and immune evasion in breast cancer patients. In mouse models, chemical sympathectomy (6-OHDA) and nerve lysates injection both accelerated tumor growth, suggesting that nerve damage promotes immune escape. Single-cell RNA sequencing (scRNA-seq) further revealed that nerve injury increased tumor-associated macrophages (TAMs) proportion by promoting TAMs proliferation and attracting macrophages. The key effector molecule of nerve lysates neurofilament light chain (NFL) was identified with the TAMs proliferation effect, and intratumoral NFL administration recapitulated the pro-tumor effects of nerve damage and perfomed the same immune-modulating effects as 6-OHDA and nerve lysates. Importantly, NFL-induced TAM enrichment and remodeling promoted CD8^+^ T cell senescence, as evidenced by transcriptomic analysis showing NF-κB pathway activation and verified with NF-κB inhibitor (BAY 11-7082) in vitro, resulting in breast cancer immune escape.

**Conclusion:**

These findings underscore the critical role of peripheral nerve injury in reshaping the interplay between TAMs and antitumor immunity, via NFL-driven NF-κB activation and T cell dysfunction. Suggesting that neuroprotection could serve as a promising strategy to restore anticancer immunosurveillance.

**Supplementary Information:**

The online version contains supplementary material available at 10.1186/s13046-025-03545-x.

## Introduction

The nervous system is a crucial regulator of the ecological niches of both healthy and malignant cells throughout the body [1]. It governs the development, homeostasis, and regeneration of organs over the lifetime and is central to the physiological functions of all organ systems, including the immune system [1, 2]. The nervous system also plays a pivotal role in cancer, where tumor development is a complex multistep process involving cell proliferation, invasion, angiogenesis, and metastasis [3–6]. Recent research has revealed close ties between the peripheral nervous system and tumor biology, particularly highlighting the significance of neurotumoral interactions within the tumor microenvironment [5, 7]. Peripheral nerves are not only instrumental in the initiation and progression of tumors, but their damage or dysfunction can also significantly influence cancer malignancy [7, 8].

Damage to peripheral nerves has been correlated with the progression of various types of cancers, including breast, lung and pancreatic [9–12]. This connection may be mediated through multiple mechanisms; for instance, neurotransmitters and neuropeptides released by peripheral nerves may directly promote the growth and invasion of cancer cells or alter the behavior of immune cells by modulating the tumor microenvironment [12]. Additionally, peripheral nerve damage can lead to pain and inflammatory responses, which may also be associated with the malignant progression of tumors [11, 13]. In the case of the breast, previous studies have identified significant adrenergic sympathetic innervation through immunohistochemical studies and retrograde transneuronal viral tracing techniques and have suggested that the sympathetic nervous system promotes the progression of pancreatic and breast cancers [14–16]. However, one study [17] reported that tumor growth and metastasis were accelerated in KIC mice, which is a spontaneous pancreatic cancer transgenic model, with peripheral nerves ablated using 6-OHDA, a result consistent with our findings in immunocompetent breast cancer-bearing mice using the same treatment. The precise mechanisms by which the intratumoral nerves affect tumor progression require further investigation.

Given that the functions of the immune system within the tumor microenvironment are also directly influenced by the nervous system, the interplay among neuro-immune-tumor components forms a complex network that affects tumor growth, inflammatory responses, and immune surveillance. Although some aspects of these interactions have been illuminated by research, a comprehensive understanding of the interplay between tumors, nerves, and the immune system is still in its infancy.

The innervation of the breast is primarily derived from somatic sensory nerves and autonomic nerves that accompany blood vessels. Specifically, the nipple and areola are richly innervated by somatic sensory nerves, while the breast parenchyma is predominantly innervated by autonomic nerves, specifically sympathetic nerves. Sympathetic nerves play a key role in regulating breast gland secretion and smooth muscle contraction [18]. Interestingly, cancerous changes in the breast typically occur not in the nipple-areola complex but within the breast parenchyma. This anatomical distinction highlights the importance of exploring the role of sympathetic nerve injury in tumor progression, particularly in the context of breast cancer. This study aimed to investigate how peripheral nerve damage affects breast cancer progression, particularly, how the destruction of peripheral nerves affects the advancement of breast cancer via immune modulation.

## Materials and methods

### Human blood samples

All human studies were approved by the Medical Ethics Committee of Sun Yat-sen Memorial Hospital, Sun Yat-sen University (Approval No. SYSKY-2024-1135-01). Written informed consent was obtained from healthy volunteers in compliance with the Declaration of Helsinki.

For participant recruitment and blood collection: Approximately 20 mL of peripheral blood was collected from healthy volunteers who met the following inclusion criteria: (1) females aged 18–60 years; (2) body weight ≥ 45 kg; (3) hemoglobin levels ≥ 120 g/L; (4) normal vital signs. Exclusion criteria included: (1) history of infectious diseases including but not limited to HIV, hepatitis B, hepatitis C, or syphilis; (2) current or recent (within 4 weeks) use of immunosuppressive medications or antibiotics; (3) active autoimmune disorders; (4) pregnancy or breastfeeding; (5) history of malignancy within the past 5 years; (6) recent vaccination (within 4 weeks); (7) blood donation within the past 8 weeks. All blood samples were collected using standard phlebotomy procedures under sterile conditions and were immediately processed for peripheral blood mononuclear cell (PBMC) isolation.

For the isolation of peripheral blood mononuclear cells (PBMCs), 20mL of fresh blood was layered onto 15 mL of Ficoll-Paque in a 50 mL centrifuge tube and centrifuged at 450 g for 20 min. The PBMC layer was aspirated and diluted to 50 mL with physiological saline and centrifuged at 400 g for 5 min. The supernatant was discarded, and the cells were resuspended in physiological saline, followed by two additional washes at 350 g for 5 min each.

### Animal experiments

Female C57BL/6 and NOD-SCID mice (6–8 weeks old) were obtained from the Jackson Laboratory and housed in groups of five per cage under a 12-hour light/dark cycle with ad libitum access to food and water. All animal experiments were approved by the Animal Ethics Committee of Sun Yat-sen Memorial Hospital (Approval No. AP20240248). PY8119 (5 × 10⁵) or EO771 (1 × 10^6^) cells were orthotopically injected into the inguinal fat pad beneath the fourth nipple pair of C57BL/6 or NOD-SCID mice. For chemical sympathectomy, mice were anesthetized with 1% sodium pentobarbital and administered 6-hydroxydopamine (6-OHDA; #S5324, 2 mg/20 g body weight, i.p.) or saline (vehicle control) on the day of tumor implantation and again on day 5 to prevent reinnervation. For NFL treatment, NFL (CUSABIO, CSB-YP015688MO, 200 µg/200 g body weight) or PBS (control) was mixed with the tumor cell suspension prior to implantation. After tumor establishment, a single intratumoral injection (10 µL) was administered. For nerve lysate treatment, the procedure was identical to that of the NFL protocol, but normal saline was used as the control. In compliance with ethical guidelines, tumor size was restricted to ≤ 2000 mm³. Mice were monitored daily and euthanized if tumors exceeded this limit or if body weight loss exceeded 20%.

### Cell lines

PY8119 (#CRL-3278), EO771(#CRL-3461), RAW264.7 (#TIB-71) and THP1 (#TIB-202) cells were purchased from the American Type Culture Collection. All cell lines were cultured in Dulbecco’s Modified Eagle’s Medium (DMEM) with 10% fetal bovine serum, and maintained at 37 °C in a humidified incubator with 5% CO₂. RAW264.7 and THP1 cells were specifically treated with NFL at final concentrations of 50 ng/mL for 24 h. All cell lines were tested monthly for mycoplasma contamination, and new cells were thawed from frozen stocks after 12 weeks in culture.

### Preparation of nerve lysate

Following euthanasia, the sciatic nerve was exposed via a diagonal incision above the femoral head, excised, and placed in ice-cold saline at 100 µL per 20 mg of tissue. Nerve lysate was prepared by three freeze-thaw cycles (30 s in liquid nitrogen, 1 min thawing), similar to previously described protocol [19].

### Mouse primary cell isolation

Subcutaneous tumors were minced into small fragments to facilitate enzymatic digestion. The fragments were then incubated in RPMI-1640 medium (Invitrogen) supplemented with 0.2 mg/mL collagenase I (Worthington, LS004196) and 0.2 mg /mL collagenase IV (Worthington, LS004188), and 0.01 mg/mL DNase I (Roche, 10104159001) and 2% fetal bovine serum at 37 °C to obtain a single-cell suspension. The resulting cell suspension was filtered through a 70 μm cell strainer to remove clumps and debris.

For CD11b⁺ monocytes and CD8⁺ T cell collection, mouse spleens were harvested and cleared of surrounding adipose and connective tissues. After thorough grinding and passage through a 70 μm cell strainer, the collected cells were resuspended in 4 mL Mouse Lymphocyte Separation Medium (Dakewe, DKW33-R0100). PBS (1 mL) was carefully layered on top, and the suspension was centrifuged at 800 × g for 30 min without brake at room temperature. The resulting cells were resuspended in RPMI-1640 complete medium at 1 × 10^8^ cells /mL. CD11b⁺ cells and CD8⁺ T cells were then separated using the EasySep™ Mouse CD11b Positive Selection Kit II (stemcell, 18970) and REAlease^®^ CD8a MicroBead Kit, mouse (Miltenyi, 130-126-707), respectively, following the manufacturer’s instructions, and immediately used for downstream functional assays and experimental analyses.

### ScRNA seq

Immune cells (CD45^+^) were isolated from C57BL/6 subcutaneous tumors using MACS (Miltenyi, 130-052-301). For nerve lysate or NS treated group, total 20,451 cells were used for analysis, while 20,565 cells were selected in NFL or PBS treated group. Single-cell RNA-Seq libraries were prepared using SeekOne^®^ Digital Droplet Single Cell 3’ library preparation kit (SeekGene, Catalog No. K00202). The libraries were then sequenced on DNBSEQ-T7 platform with PE150 read length. Dimensionality reduction and clustering were conducted using the Seurat V5 R package.

### Generation of bone marrow-derived macrophages (BMDMs) and monocyte-derived macrophages (MDMs)

Bone marrow cells were isolated from C57BL/6 mice by flushing the femoral and tibial cavities with cold RPMI 1640 medium, repeating the process until no red color remained. The medium containingbone marrow cells were filtered through a 70 μm filter into a 15 mL centrifuge tube and centrifuged at 350 g for 5 min. Red blood cells were lysed using a lysis buffer and washed with PBS. For generation of BMDMs, the bone marrow cells were cultured in RPMI 1640 induction medium with 10% FBS and 5 ng/ml M-CSF (Novoprotein, CB34) for 7 days to allow maturation. During the culture process, half of the medium was replaced on day 3 and completely replaced on day 5.

For generation of MDMs, monocytes were isolated from human PBMC using CD14 magnetic beads (Miltenyi, 130-050-201) and resuspended in DMEM containing 20 ng/mL M-CSF (Novoprotein, C417) and 10% FBS. The cells were seeded in a 24-well plate at 5 × 10⁵ cells per well and cultured for 7 days, with medium changes every 3 days to induce differentiation into monocyte-derived macrophages (MDMs). For co-culture experiments, MDMs were seeded at 2 × 10⁴cells/well in 24-well plates with direct (no Transwell) and indirect (Transwell) co-culture systems.

### Flow cytometry

To distinguish live and dead cells, freshly prepared single cell suspensions were incubated with Fixable Viability Stain (BD, 565388) at room temperature for 15 min. For membrane staining, antibodies were diluted in staining buffer (PBS supplemented with 2% FBS) and incubated on ice for 30 min. The antibodies used included FITC anti-mouse CD45 (BioLegend, 103108), Brilliant Violet 605™ anti-mouse/human CD11b (BioLegend, 101237), PE anti-mouse F4/80 (BioLegend, 123109), PE anti-mouse CD4 (BioLegend, 100407), APC anti-mouse CD8a (BioLegend, 100711), APC anti-human CD8 (BioLegend, 980908). For intracellular staining, cells were then fixed and permeabilized using an Intracellular Fixation/Permeabilization Buffer Kit (Elabscience, E-CK-A109) according to the manufacturer’s instructions. Brilliant Violet 421™ anti-human/mouse Granzyme B (BioLegend, 396413) or anti-Ki-67 (BioLegend, 151225) were added to permeabilization buffer and stained for 30 min at room temperature. Data acquisition was performed on a CytoFLEX flow cytometer and analyzed by FlowJo software.

### Immunohistochemistry

Formalin-fixed, paraffin-embedded 5 μm sections of mouse cancer tissue were cuted with a microtome, mounted on adhesive slides and dried at 60 °C. For immunohistochemistry, heat-induced antigen retrieval was performed at pH 9 (ZLI-9069), 100 °C for 10 min then washed with PBS and subsequently blocked with 5% BSA in PBS for 1 h at room temperature. Subsequently, the sections were incubated with primary antibodies against NFL (proteintech, 60189, 1:500) overnight at 4 °C. Anti-mouse HRP (ZB-5305) was utilized asdetection method and incubated for 60 min with the primary antibody. The reaction was visualized with diaminobenzidine (DAB, ZLI-9017) and slides were counterstained with haematoxylin. The slides were imaged using a slide scanner (KFBIO/KF-FL-400, China), and images were analyzed using ImageJ software by manually selecting positively stained regions.

### Immunofluorescence

For immunostaining of cultured cells, cells were fixed with paraformaldehyde and permeabilized for 15 min using 0.1% Triton X-100. Nonspecific binding was blocked by incubating the samples in PBS containing 5% BSA for 1 h at room temperature. Subsequently, cells were incubated overnight at 4 °C with Ki67 primary antibodies (Starter, S0B2332, 1:200). The next day, the samples were washed with PBS and processed with the appropriate secondary antibody detection method. For tumor tissue analysis via multiplex immunofluorescence, samples were first paraffin-embedded and sectioned at 4 μm thickness. Sections were deparaffinized, rehydrated, and subjected to antigen retrieval by incubating the slides in 0.01 M EDTA buffer (pH 8.0) following the manufacturer’s instructions for the kit (Absin, abs50014). Endogenous peroxidase activity was blocked by incubating the slides in PBS containing 3% H₂O₂ for 15 min, followed by 5% BSA blocking for 1 h. Sections were incubated overnight at 4 °C or for 2 h at room temperature with specific primary antibodies, including Cleaved Caspase-3 (CST, 9661, 1:400), pan Cytokeratin (Abcam, ab7753, 1:200), PGP9.5 (abclonal, A19101, 1:500), GZMB (Affinity, AF0175, 1:200), CD8 (Starter, S0B0034, 1:500), F4/80 (Starter, S0B0227, 1:500), and Ki67 (Starter, S0B2332, 1:500). PD1 (Abclonal,1:100), Eomes (Proteintech, 83945, 1:500), Tim3 (Proteintech, 60355, 1:400), Lag3 (Proteintech, 16616, 1:500), Tox (Abcam, AB237009, 1:300) along with IL-2 (Proteintech, 60306, 1:400), IFNγ (Affinity, DF6045, 1:100) and perforin (Abclonal, A0093, 1:100). Images were acquired on KF-FL-400 scanning system (KFbio, China). Quantitative colocalization was performed with the HALO AI Colocalization Module (Indica Labs). For tumor nerve staining, samples were sectioned at 15 μm thickness, imaged with a high speed spinning disk confocal microscope (Andor DragonflyCR-DFLY-202-40), and images were analyzed with Imaris (Bitplane) and ImageJ.

### RNA extraction and quantitative PCR analysis

RNA was extracted from cells using the Super FastPure Cell RNA Isolation Kit (Vazyme, RC102-01). The isolated RNA was quantified using a Nanodrop 2000 (Thermo Fisher).

cDNA was synthesized using the Fast First-Strand cDNA Synthesis Mix for RT (with dsDNase) kit (GOONIE, 500 − 101) according to the manufacturer’s instructions. qPCR was performed using Fast Taq SYBR Green qPCR Mix (GOONIE, 500 − 100) on the LightCycler 480 (Roche), with GAPDH as the normalization gene. mRNA expression analysis was conducted using the 2-ΔΔCt method. The primers used in RT-PCR: TNF-α (CCTGTAGCCCACGTCGTAG / GGGAGTAGACAAGGTACAACCC), IL-6 (CTGCAAGAGACTTCCATCCAG / AGTGGTATAGACAGGTCTGTTGG), and GAPDH (CTGCACCACCAACTGCTTAG / GTCTGGGATGGAAATTGTGA).

### Transwell migration assay

Transwell inserts (8 μm pore size) were placed in a 24-well plate. One day prior to seeding, cells were cultured in serum-free medium for 24 h to induce starvation. On the day of the assay, 500 µL of complete medium containing nerve lysate or NFL was added to the lower chamber. Subsequently, 5 × 10^5^ cells were seeded into the upper chamber of the Transwell insert and incubated for 48 h at 37 °C in a 5% CO₂ incubator. After incubation, the Transwell insert was removed and residual medium was gently aspirated. Wash the insert three times with PBS. Fix the cells with 4% paraformaldehyde at room temperature for 15 min. Following another three PBS washes, cells were stained with 0.1% crystal violet solution at room temperature for 5 minutes, washed three more times with PBS to remove excess crystal violet, and any non-migrated cells were carefully wiped from upper chamber with a moistened cotton swab. Finally, the insert was allowed to air-dry prior to assessment.

### Proliferation test

THP1 cells were seeded into a 96-well plate at a density of 3 × 10⁴ cells per well and allowed to acclimate for 24 h in RPMI 1640 medium supplemented with 10% FBS. The cells were then treated with NFL (50ng/ml) for 24 h, with complete medium containing NFL serving as the blank control. After treatment, 10 µL of CCK8 reagent (NCM Biotech, C6005) was directly added to each well without removing the culture medium. The plate was incubated for 1 hourat 37 °C in a 5% CO₂ incubator. Absorbance at 450 nm was measured using a microplate reader. All samples were measured in triplicate. The proliferation rate was calculated using the formula: Proliferation Rate = [(Treatment Group OD − Blank Group OD) ÷ (Control Group OD − Blank Group OD)] × 100%.

### Co-culture of macrophages with NFL and CD8^+^ T cells

For direct and indirect co-culture systems, macrophages and CD8^+^ T cells were plated in 24-well plates either in direct contact or separated by 3-µm pore transwell inserts. MDM were seeded at a density of 2 × 10^4^ cells per well in the lower chamber, followed by the addition of NFL (100 ng/mL) or PBS. Isolated CD8^+^ T cells were then seeded into the upper chamber of the Transwell insert at a density of 1 × 10^6^ cells/mL. Parallel direct co-culture experiments were performed. The co-culture system was incubated in a humidified incubator at 37 °C with 5% CO₂ for 48 h. After the co-culture period, cells were harvested from the upper chambers for further analysis.

### ELISA

The IFN-γ ELISA Kit (AF02182) and the NFL ELISA Kit (AF030415) were utilized. For neurofilament light chain (NFL) in tumor tissue after intraperitoneal injection of 6-OHDA or normal saline, tissues were washed with ice-cold PBS (0.01 M, pH 7.4), weighed, minced, homogenized in ice-cold PBS (1:9 w/v), centrifuged at 4 °C, 5000 g for 5 min, and the supernatant was collected. Sample integrity was maintained by centrifugation (10,000 × g, 10 min) and storage at − 80 °C prior to analysis. The standards and samples were processed with capture antibodies, biotinylated detection antibodies, and streptavidin-HRP, followed by TMB development and 450 nm absorbance reading. Both assays included blank, negative, and positive controls.

### Cell senescence detection

CD8^+^ T cell senescence in tumor tissues was detected by isolating primary cells from tumor samples and labeling them with the Senescence-Tracker™ Near-infrared probe (Beyotime, C0603) following the manufacturer’s protocol. Briefly, cells were seeded at 1 × 10⁶ cells/mL in 12-well plates (1mL each well) containing RPMI-1640 medium with 10% FBS, incubated with the 1:1000 diluted probe for 30 min at 37 °C in 5% CO₂, and protected from light for 48 h. After washing twice with PBS to remove unbound probe, cells were stained with FVD780 viability dye and anti-CD8 antibody for 24 h, then resuspended in 500 µL PBS for flow cytometry analysis using a CytoFLEX instrument, with β-galactosidase activity serving as the senescence marker.

## Result

### Peripheral nerve injury correlates with poor prognosis and promotes breast cancer growth

**Fig. 1:**
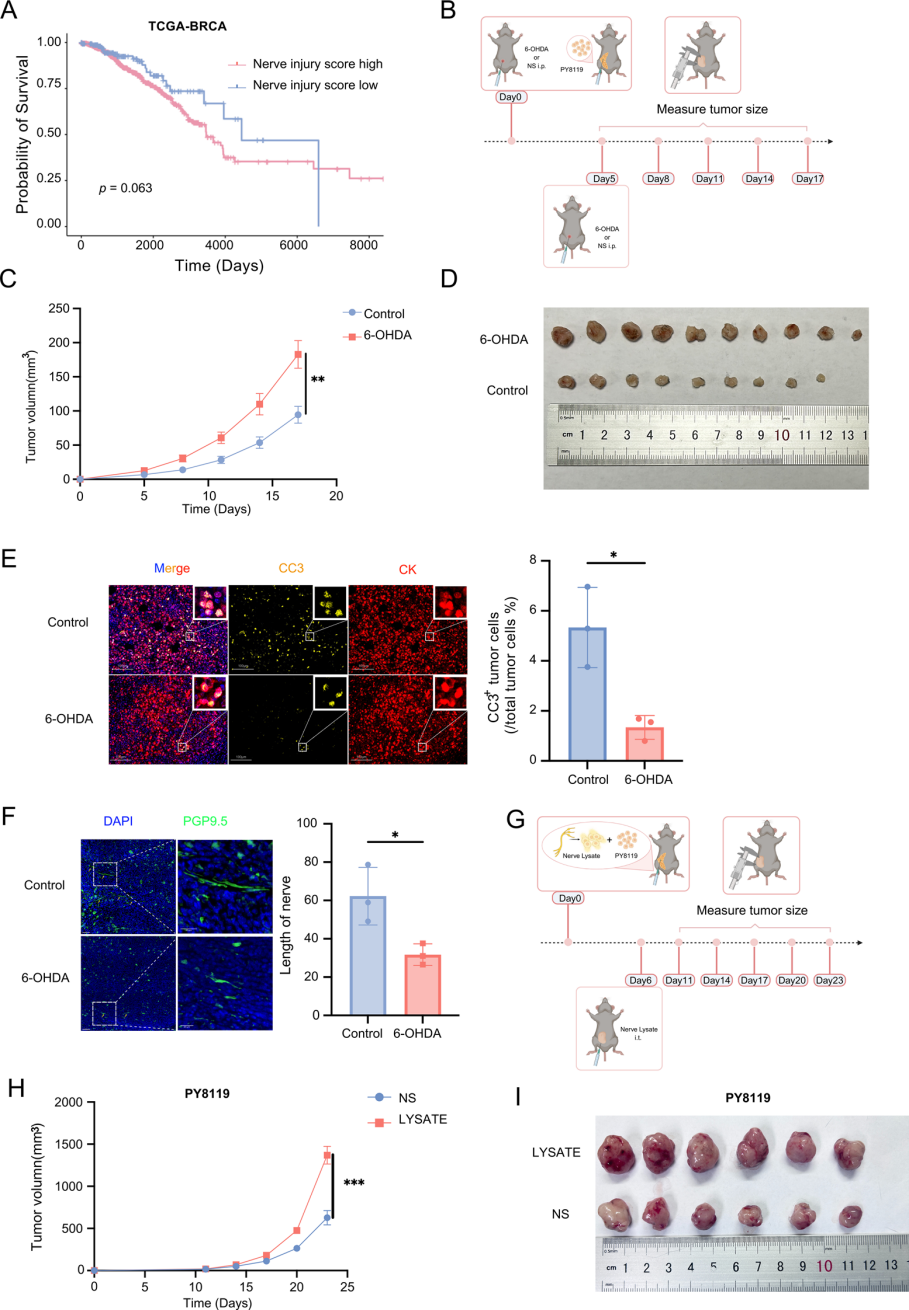
Peripheral neural injury correlated with poor prognosis and promoted breast cancer growth. (**A**) Overall survival curves for breast cancer patients with high or low degree of nerve injury in the TCGA-BRCA cohort. (**B**) Schematic of 6-OHDA treatment in a PY8119 tumor mouse model. (**C**) Tumor growth curve of PY8119 tumors upon treatment with 6-OHDA or normal saline in C57BL/6 mice. (*n*_control_ = 9, *n*_6 − OHDA_=10). (**D**) The image of tumors from the control and 6-OHDA treatment groups in C57BL/6 mice. (**E**) Representative immunofluorescence images showing Cleaved Caspase-3 (CC3) and panCK staining in tumor tissues from mice treated with 6-OHDA or normal saline. Right panel shows the proportion of dead tumor cells, quantified by CC3 and panCK co-staining. (*n* = 3 per group). (**F**) Representative immunofluorescence images showing PGP9.5 staining in tumor tissues from mice treated with 6-OHDA or normal saline(left), the right panel shows the quantification of length of nerve in two groups. (*n* = 3 per group). (**G**) Experimental design of nerve lysate treatment in a PY8119 tumor mouse model on C57BL/6 background. (**H**) Tumor growth curve of PY8119 tumor upon treatment with nerve lysate or normal saline in C57BL/6 mice. (*n* = 6 per group). (**I**) The image of tumors from the normal saline and nerve lysate treatment groups in C57BL/6 mice. Data in (**C**) and (**H**) are presented as mean ± SEM, data (**E**) and (**F**) is presented as mean ± SD. Data in (**E**) is statistic by HALO software, statistical significance was determined by two-tailed student’s t-test. *p < 0.05, **p < 0.01, ***p < 0.001, ****p < 0.0001. I.p.: Intraperitoneal injection

To investigate whether the extent of nerve injury correlates with patient prognosis, we used the GOBP_NEURON_APOPTOTIC_PROCESS gene set from the GSEA database and stratified patients with TCGA-BRCA into high- and low- expression groups based on the overall expression levels of this gene set. We plotted overall survival (OS) curves for both groups. Survival analysis revealed that breast cancer patients with higher nerve injury levels had a significantly poorer prognosis (Fig. 1A), suggesting that nerve injury may contribute to tumor progression.

6-OHDA treatment could significantly reduce the abundance of sympathetic nerve fibers in the mammary gland [20]. It enters neurons through dopamine or norepinephrine transporters [21], generating reactive oxygen species (ROS) that disrupt cellular metabolism [22] and lead to neuronal death, as a common method for modeling nerve injury [23–25]. To further explore the role of nerve injury in tumor progression, we intraperitoneally administered 6-OHDA to C57BL/6 mice (Fig. 1B). PY8119, a spontaneous mammary tumor cell line [26], was used for fat-pad tumor implantation. Upon monitoring tumor growth, we found that tumors in the 6-OHDA-treated group grew significantly faster than those in the sham group (Fig. 1C-D). Consistent with this observation, immunofluorescence staining and statistical analyses revealed a significant decrease in the proportion of apoptotic tumor cells in the 6-OHDA group (Fig. 1E). To evaluate whether 6-OHDA effectively induced nerve injury in the breast, we performed PGP9.5 immunofluorescent staining on breast tumor tissue samples from mice treated with or without 6-OHDA. The results demonstrated that intraperitoneal administration of 6-OHDA significantly reduced nerve fiber density in the breast, successfully establishing a model of nerve injury (Fig. 1F). Furthermore, to directly observe the effect of nerve damage on tumor progression, we obtained sciatic nerves from C57BL/6 mice and added a certain proportion of physiological saline, followed by repeated freezing and thawing with liquid nitrogen to obtain a nerve lysate, which was injected into the tumor to simulate nerve damage. Using the intratumoral nerve lysate model (Fig. 1G), we observed that tumor growth was significantly faster in the nerve lysate group than in the saline-injected control group (Fig. 1H and I), consistent with the effect of 6-OHDA treatment. Additionally, to validate these findings across breast cancer subtypes, we conducted additional experiments using murine EO771 cells (luminal-like characteristics) [27]. 6-OHDA and nerve lysate (NL) treatments in EO771 tumors recapitulated the Py8119 model results, showing consistent patterns of accelerated tumor growth (Figure S1A-B).

These findings indicate that nerve injury accelerates tumor progression, and that nerve integrity plays an important role in curbing the malignant progression of tumors.

### Peripheral nerve destruction restrains anti-tumor immunity

**Fig. 2:**
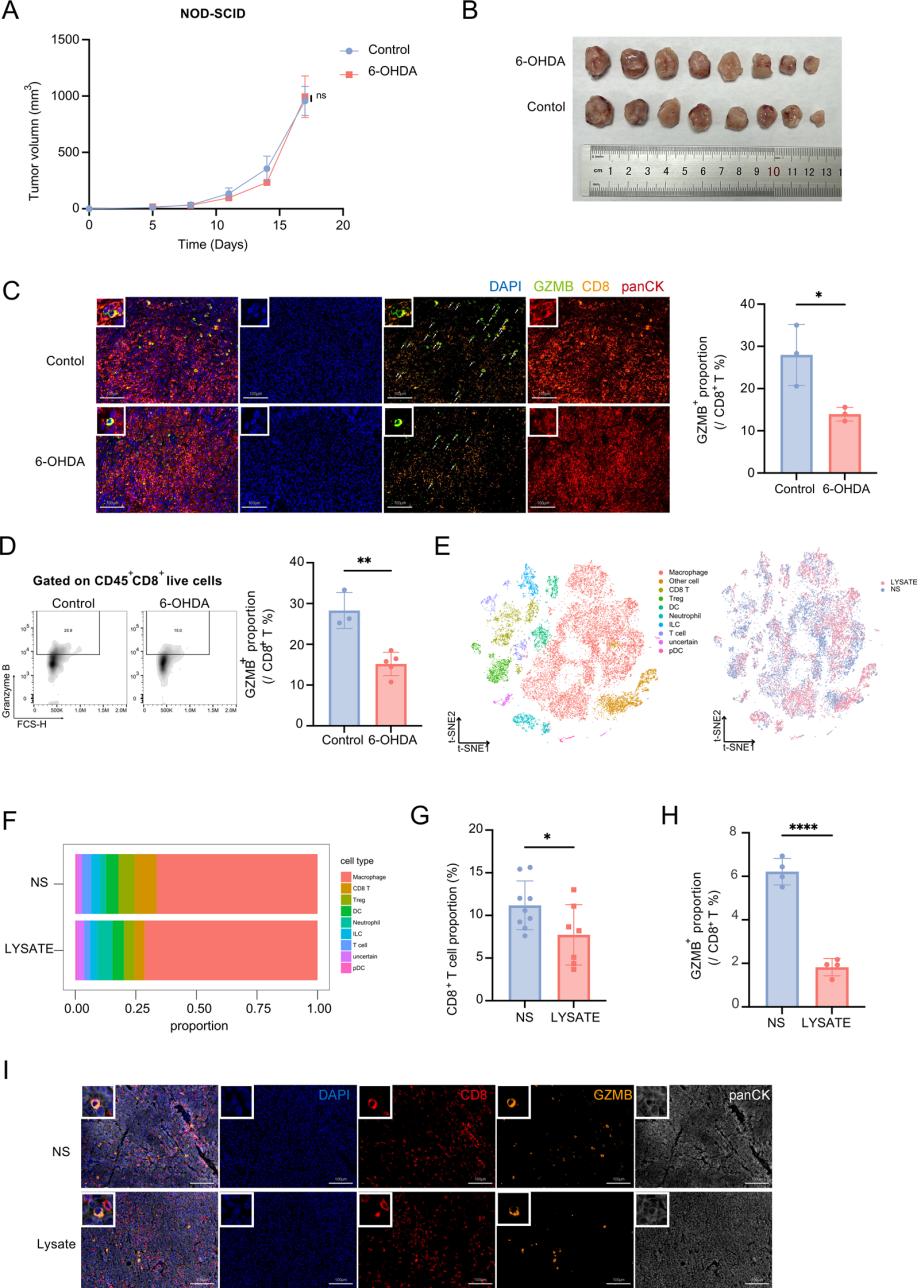
Peripheral nerve destruction restrained antitumor immunity. (**A**) Area under the curve of tumor size in NOD-SCID mice upon treatment with 6-OHDA or normal saline. (*n* = 8 per group). (**B**) The image of tumors from the control and 6-OHDA treatment groups.in NOD-SCID mice. (**C**) Representative immunofluorescent images of Granzyme B (GZMB), CD8, and panCK staining in tumor tissues from mice treated with 6-OHDA or normal saline (left). Right panel shows the proportion of GZMB^+^ cells among total CD8^+^ T cells. (*n* = 3 each group). (**D**) Percentage of GZMB^+^ cells among CD45^+^CD8^+^ T cells, as measured by flow cytometry. (*n*_Control_ = 3, *n*_6 − OHDA_ = 6. (**E**) t-SNE map of immune cell populations following treatment with nerve lysate or normal saline (left). Right panel shows t-SNE map of immune cell population distributions, grouped by treatment. (**F**) Proportional distribution of immune cells in tumor tissues from mice treated with LYSATE or NS. (**G**) Percentage of CD8^+^ T cells, as measured by mIHC, statistic by HALO software. (**H**) Proportion of GZMB^+^ cells within total CD8^+^ T cells in the LYSATE and NS treatment groups, as measured by mIHC, statistic by HALO software. (**I**) Representative immunofluorescent images of (**G**) and (**H**). Data in (**A**) are presented as mean ± SEM, while other data are presented as mean ± SD. Data in (**G**) and (**H**) are statistic by HALO software. Statistical significance was determined using a t-test. *p < 0.05, **p < 0.01, ***p < 0.001, ****p < 0.0001

Tumor development cannot be separated from immune system regulation. When antitumor immunity is suppressed, tumors often undergo malignant progression [28]. To determine whether nerve injury impacts antitumor immunity, we compared tumor growth in immunodeficient (NOD-SCID) and immunocompetent (C57BL/6) mice after 6-OHDA treatment. While tumor growth increased in C57BL/6 mice (Fig. 1C-D), no difference was observed in NOD-SCID mice (Fig. 2A and B), indicating that nerve injury-driven tumor progression requires an intact immune system. This lack of difference in the immunodeficiency model led us to hypothesize that nerve injury may affect antitumor immunity, which could explain the observed differences in immunocompetent mice.

CD8^+^ T-cells are believed to play a major role in antitumor immunity. To further explore whether nerve damage is directly related to CD8^+^ infiltration within the tumor, we analyzed the relationship between nerve injury severity and CD8^+^ T cell infiltration in breast cancer patients using the TCGA-BRCA dataset and stratified them into high and low nerve damage groups according to the severity of nerve damage. Then, the infiltration score of CD8^+^ T cells was evaluated using CIBERSORT. Correlation analysis revealed a significant reduction in CD8^+^ T cell infiltration in patients with higher levels of nerve damage (Figure S2A and Figure S2B). This indicates that nerve injury could negatively affect CD8^+^ T cell-mediated anti-tumor immunity in patients with breast cancer. In anti-tumor immunity, CD8^+^ T cells typically exert their anti-tumor effects by releasing molecules such as granzyme B (GZMB). To assess the anti-tumor efficacy of CD8^+^ T cells, immunofluorescence and flow cytometry were used to evaluate this phenomenon in an animal model.

Immunofluorescence analysis of tumor tissues from 6-OHDA-treated and sham-treated mice supported this hypothesis. The 6-OHDA group exhibited a significantly lower proportion of GZMB^+^ CD8^+^ T cells (Fig. 2C). Flow cytometry confirmed these findings, revealing fewer GZMB^+^ CD8^+^ T-cells in the 6-OHDA-treated group versus controls (Fig. 2D). To further characterize CD8^+^ T cell dysfunction, we analyzed nerve injury-associated changes within CD8^+^ T cell exhaustion, including PD1, Eomes, Tim3, Lag3, KLRG1, Tox, as well as the cytokines IL-2, IFNγ, and Perforin via TCGA-BRCA dataset (Figure S3A).

To obtain more comprehensive information on antitumor immunity during nerve injury, especially changes in CD8^+^ T cells, we next performed single-cell RNA sequencing on CD45^+^ immune cells isolated from the tumors of animals treated with either nerve lysate or normal saline intratumoral injection (Fig. 1H and Figure S2C). After quality control (Figure S2D), the 20,451 cells were clustered into 10 distinct populations and annotated based on high-variance genes (Figure S3E and 2E). A stacked bar plot showing the proportional distribution of these immune cell clusters revealed a significant decrease in the proportion of CD8^+^ T-cells in the nerve lysate group (Fig. 2F). This change was also confirmed through CD8 staining of our tissue sections (Fig. 2G and I). Additionally, we analyzed both exhaustion markers and functional markers in tumor-infiltrating CD8^+^ T cells through single-cell RNA sequencing and immunofluorence staining of tumor section (Figure S3B-C) to comprehensively evaluate the functional state of CD8^+^ T cells. Changes of GZMB were further validated by immunofluorescence staining and statistical analyses (Fig. 2H-I), confirming that nerve lysate treatment impaired CD8^+^ T-cell proliferation and cytotoxic function.

Together, these findings suggest that when nerves are damaged, antitumor immunity is suppressed, particularly with the loss of CD8^+^ T-cell function.

### Nerve injury mediated macrophage proliferation and migration, inducing immune suppression

**Fig. 3:**
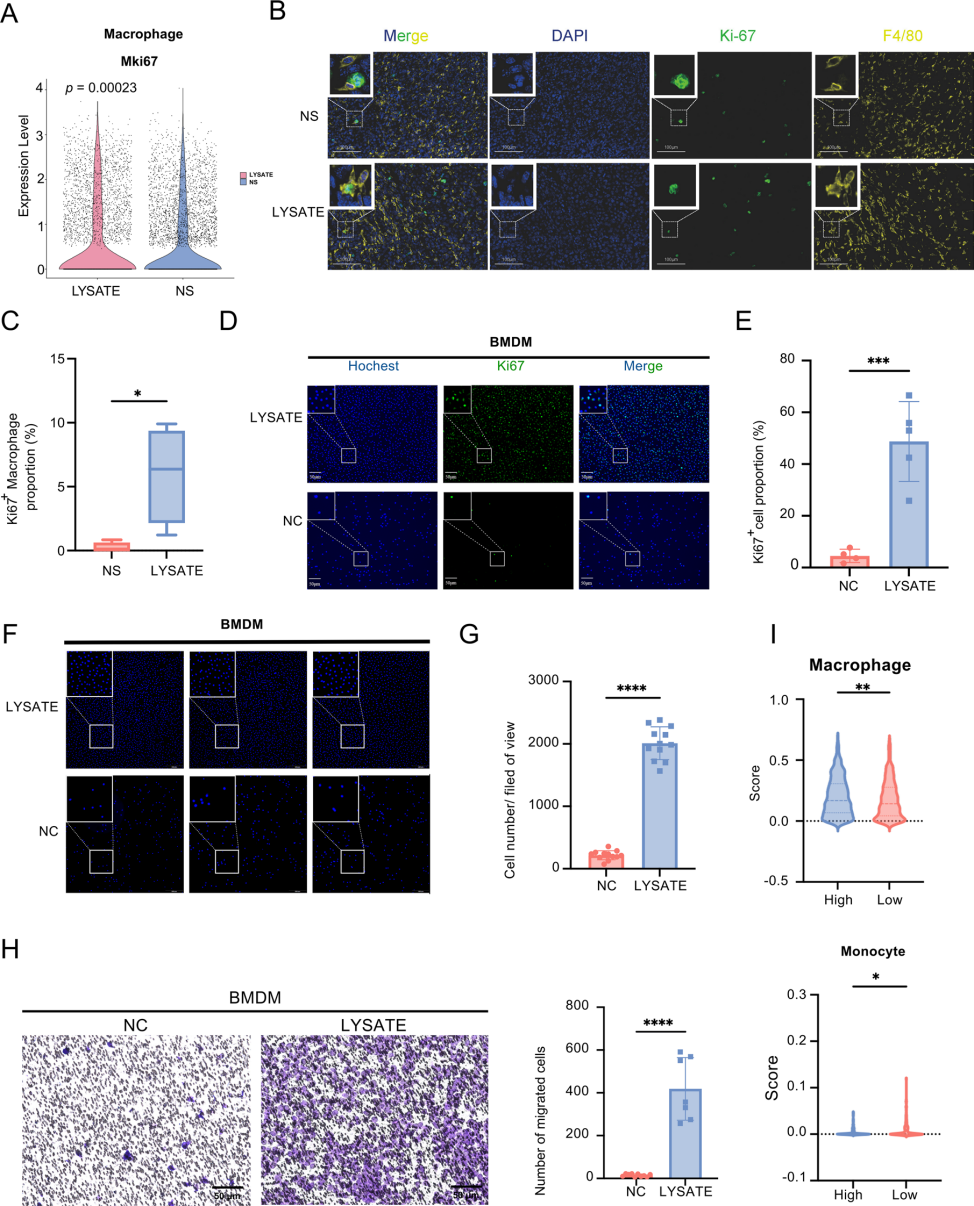
Nerve injury induces macrophage proliferation and migration. (**A**) Expression of Mki67 in macrophages following treatment with nerve lysate or normal saline (NS). (**B**) Representative immunofluorescent images of Ki67 and F4/80 staining in tumor tissues from mice treated with nerve lysate or normal saline (NS). (**C**) The proportion of Ki67^+^F4/80^+^ cells among all F4/80^+^ cells, (*n* = 3 each group). (**D**) Representative immunofluorescent images of Hochest and Ki67 staining in bone marrow-derived macrophages (BMDMs). (**E**) The proportion of Ki67^+^ cells. (**F**) Representative immunofluorescent images of DAPI staining in bone marrow-derived macrophages (BMDMs). (**G**) The cell number in each fileld of view. (**H**) Representative Transwell migration images (left) and statistical results (right) of BMDMs treated with NC or LYSATE. (**I**) Macrophage (up) and monocyte (down) infiltration scores in TCGA-BRCA patients with high or low degrees of nerve injury. Data in (**C**) is presented as median (min–max), all other data are presented as mean ± SD. Statistical significance was determined using a t-test. ***p < 0.001, ****p < 0.0001

To investigate the effect of nerve injury on macrophage proliferation, we analyzed the expression of Mki67, a marker of cell proliferation, in the nerve lysate-treated group. At the single-cell sequencing level, the Vln Plot showed that macrophages in the nerve lysate-treated group expressed higher levels of Mki67 (Fig. 3A). Besides, immunofluorescence staining and statistical analysis of tumor tissue sections confirmed that a higher Ki67^+^ proportion of macrophages were present in the nerve lysate-treated group (Fig. 3B-C). In addition, immunofluorescence staining, flow cytometry and statistical analysis of tumor tissue sections confirmed that a higher proportion of macrophages were present in the nerve lysate-treated group (Figure S4A-D).

In vitro, we treated mouse bone marrow-derived macrophages (BMDMs) with nerve lysate and observed a significant proliferative effect. This was demonstrated by immunofluorescence staining of Ki-67 (Fig. 3D-E), and cell counting for proliferation in BMDMs (Fig. 3F-G). Furthermore, Transwell migration assays with 8 μm pore size chambers demonstrated that nerve lysate treatment enhanced the migratory ability of BMDMs (Fig. 3H).

An increase in tumor-associated macrophages (TAMs) is generally indicative of weaker antitumor immunity and immune evasion [29]. Macrophages that infiltrate tumor tissues mainly originate from peripheral blood monocytes [30]. Our analysis of macrophage and monocyte proportions in the high and low nerve damage groups of patients with breast cancer using the CIBERSORT algorithm showed similar trends, with a higher proportion of TAMs in the high nerve damage group (Fig. 3I).

### Nerve injury promoted breast cancer immune suppression via NFL

**Fig. 4:**
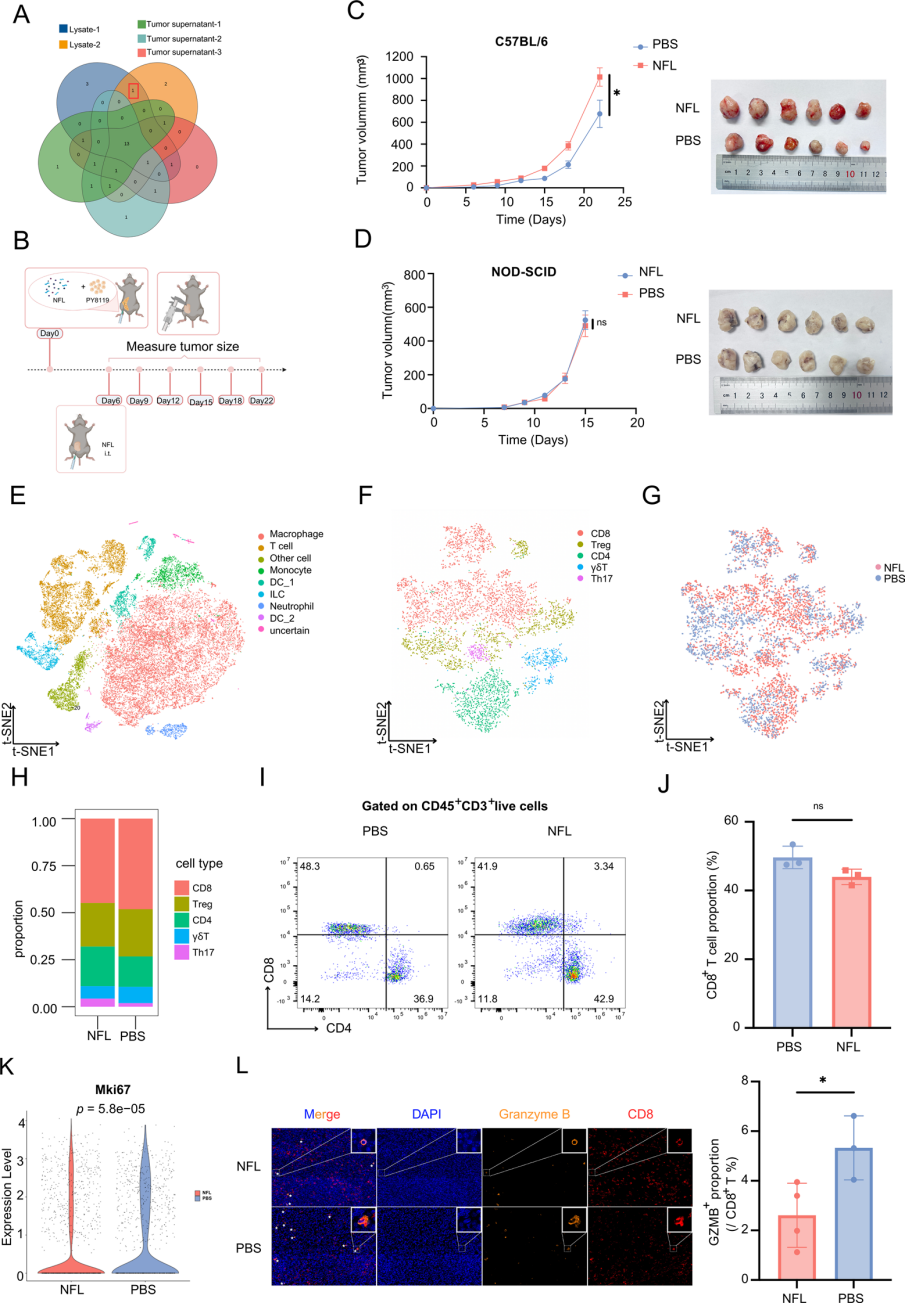
Nerve Injury Promoted Breast Cancer Immune Suppression via NFL. (**A**) The Venn diagram analysis of the top 20 high-scoring proteins (> 10 kDa) from 2 neural lysate samples and 3 tumor supernatant samples identified neurofilament light chain (NFL) as the unique intersection protein present in nerve injury conditions but absent in tumor microenvironments. (**B**) Experimental design of NFL treatment in a PY8119 tumor mouse model on C57BL/6 background. (**C**) Tumor growth curve(up) in C57BL/6 mice treated with NFL or PBS (up), and the image(down) of tumors from the PBS and NFL treatment group in C57BL/6 mice. (*n* = 6 per group). (**D**) Tumor growth curve(left) in NOD-SCID mice treated with NFL or PBS (left), and the image(right) of tumors from the PBS and NFL treatment groups in NOD-SCID mice. (*n* = 6 per group). (**E**) t-SNE map showing the distribution of immune cell populations, grouped by treatment. (**F**) t-SNE map of different T cell subtypes. (**G**) t-SNE map showing T cell subtype distributions, grouped by treatment. (**H**) Proportional distribution of T cells in tumor tissues from mice treated with NFL or PBS. (**I**) Representative flow cytometry plot showing percentage of CD8^+^ or CD4^+^ cells among CD45^+^CD3^+^ live cells from tumor tissues of mice treated with NFL or PBS. (**J**) A scatter plot represents the percentage of CD8^+^ T cells in (**I**), (*n* = 3 pergroup). (**K**) Violin plot showing the expression level of Mki67 in the NFL and PBS treatment groups. (**L**) Representative immunofluorescent images of Granzyme B and CD8 staining in tumor tissues from mice treated with NFL or PBS (left). Right panel shows the proportion of GZMB^+^ cells among total CD8^+^ T cells, as measured by mIHC, statistic by HALO software, (*n*_NFL_ = 4, *n*_PBS_ = 3). All data are presented as mean ± SD. Statistical significance was determined using a t-test. *p < 0.05

To further investigate whether the suppression of antitumor immunity was caused by nerve injury, we fractionated neuronal lysates by molecular weight (> 10 kDa vs. <10 kDa) and found that the > 10 kDa fraction drove macrophage proliferation (Figure S5A), suggesting a protein-mediated effect. Dectecting the high abundant protein and comparing them derived from neuronal and tumor-derived supernatants, we identified neurofilament light chain (NFL) in neuronal lysates, implicating NFL as the active component (Fig. 4A). We conducted experiments using NFL in the PY8119 mammary tumor model, after tumor formation was confirmed, intratumoral injections of NFL (200 ng/20 g) or PBS were administered, and tumor volumes were regularly measured (Fig. 4B). Our results revealed that tumors in the NFL-treated group exhibited significantly faster growth than those in the control group (Fig. 4C). Immunohistochemistry and ELISA revealed markedly elevated neurofilament light chain (NFL) within tumors after intraperitoneal 6-OHDA versus normal saline (Figure S5B), supporting the notion that nerve injury promotes tumor progression. In contrast, when we treated immunodeficient mice with NFL, there was no difference in the growth curve of the tumors (Fig. 4D).

Next, to assess the impact of NFL treatment on antitumor immunity, we isolated tumor-infiltrating immune cells and performed single-cell RNA sequencing, similar to the nerve lysate treatment. Total 20,565 cells were used for analysis. After QC (Figure S5C), a t-SNE map was drawn to identify major immune cell populations, including macrophages, T-cells, monocytes, dendritic cells (DCs), innate lymphoid cells (ILCs), and neutrophils (Fig. 4E and Figure S5D-E), with T-cells and macrophages being the predominant populations. We focused specifically on T-cells and further classified them (Fig. 4F and Figure S5F-G). Our analysis revealed a decrease in the proportion of CD8^+^ T-cells in the NFL-treated group (Fig. 4G-H), which was similar to the results of nerve lysate treatment. The proportion of CD8^+^ T cell proportion detected by flow cytometry showed similar trend (Fig. 4I-J), although this trend did not reach statistical significance. Additionally, the expression level of Mki67 was significantly reduced in CD8^+^ T cells following NFL treatment (Fig. 4K), similar to those of the lysate treatment. Immunofluorescence staining of the tumor sections further supported these findings, showing a lower proportion of GZMB^+^ CD8^+^ T-cells in the NFL-treated group than in the control group (Fig. 4L). Also, NFL treatments in EO771 tumors recapitulated the Py8119 model results, showing consistent patterns of accelerated tumor growth (Figure S5H).

Together, these results suggest that NFL treatment impairs CD8^+^ T-cell function, leading to suppressed antitumor immunity.

**Fig. 5:**
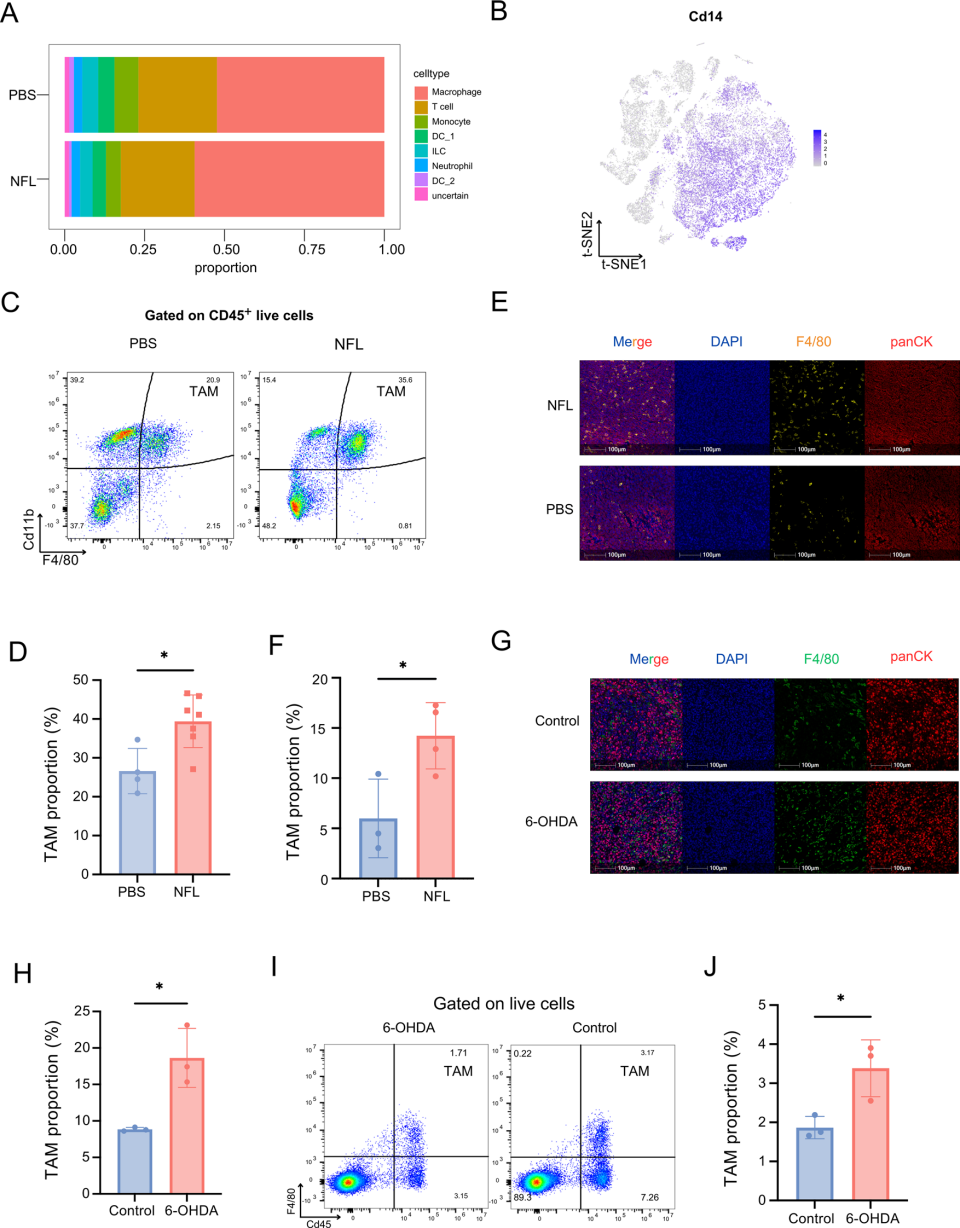
NFL treatment increased the proportion of tumor-infiltrating macrophages. (**A**) Proportional distribution of myeloid cells in tumor tissues from mice treated with NFL or PBS. (**B**) Feature plot showing the expression of *Cd14* in immune cells from tumor tissues. (**C**) Representative flow cytometry plot showing immune cells from tumor tissues of mice treated with PBS or NFL. (**D**) The proportion of TAM (CD11b^+^F4/80^+^) cells within CD45 + live cells in (**C**), (*n*_PBS_ = 4, *n*_NFL_ = 7). (**E**) Representative immunofluorescent images of F4/80 and panCK staining in tumor tissues from mice treated with NFL or PBS. (**F**) shows the proportion of F4/80^+^ cells among total cells in (**E**), statistic by HALO software, (*n*_PBS_ = 3, *n*_NFL_ = 4). (**G**) Representative immunofluorescent images of F4/80 and panCK staining in tumor tissues from mice treated with NS (control) or 6-OHDA. (**H**) The proportion of TAM (CD45^+^/F4/80^+^) cells within cells in (**G**), (*n*_Control_ = 3, *n*_6 − OHDA_ = 3). (**I**) Representative flow cytometry plot showing TAM (F4/80^+^) cells from tumor tissues of mice treated with NS (control) or 6-OHDA. (**J**) The proportion of TAM (CD11b^+^F4/80^+^) cells within live cells in (**I**), (*n*_Control_ = 3, *n*_6 − OHDA_ = 3). All data are presented as mean ± SD. Data in (**F**) and (**H**) were statistically analyzed using HALO software. Statistical significance was determined using a t-test. *p < 0.05, **p < 0.01, ***p < 0.001

As previously mentioned, single-cell RNA sequencing of tumor-infiltrating immune cells revealed the predominance of macrophages and T-cells within the tumor microenvironment (Fig. 4E). To further investigate the effect of NFL treatment on immune cell populations, we created a cell proportion chart that showed an increase in the proportion of macrophages following NFL treatment (Fig. 5A-B). Similar results were observed in the nerve lysate treatment group compared to the normal saline (NS) control group (Fig. 2F). Moreover, flow cytometric analysis of CD11b^+^F4/80^+^ double-positive macrophages confirmed a higher proportion of these cells in the NFL-treated groups. (Fig. 5C-D). Immunofluorescence analysis of the tumor tissue sections confirmed these findings, with an increased presence of macrophages in NFL-treated tumors (Fig. 5E-F). Immunofluorescence staining and quantitative analysis of tumor sections further showed a higher proportion of Ki67^+^ macrophages (F4/80^+^Ki67^+^) in the NFL-treated group (Figure S6E), consistent with the proliferative phenotype observed following nerve lysate treatment (Fig. 3B-C). Furthermore, immunofluorescence staining (Fig. 5G-H) and flow cytometry analysis (Fig. 5I-J) of the 6-OHDA-treated tumor samples also revealed an increase in the proportion of tumor-associated macrophages (TAMs) within the tumor.

Additionally, we treated human monocyte-derived macrophages (MDMs) and THP1 with or without NFL in vitro. Assessments were made using immunofluorescence staining (Figure S6A), cell counting (Figure S6B), transwell (Figure S6D) and CCK8 proliferation assays (Figure S6C). These results further support the observation that NFL induces a proliferative phenotype of macrophages.

These parallel observations across distinct subtypes underscore the broad relevance of nerve injury-induced immunomodulation in breast cancer progression.

### NFL-induced TAM enrichment promoted CD8^+^ T cell senescence

Tumor-associated macrophages accumulate in the tumor microenvironment where they suppress CD8^+^ T-cell function through various mechanisms, thereby weakening the antitumor immune response [31–33]. As previously mentioned, we observed that CD8^+^ T-cells exhibited defective proliferation and impaired anti-tumor effects in both the nerve lysate- and NFL-treated models, suggesting a state of T-cell exhaustion or senescence.

**Fig. 6:**
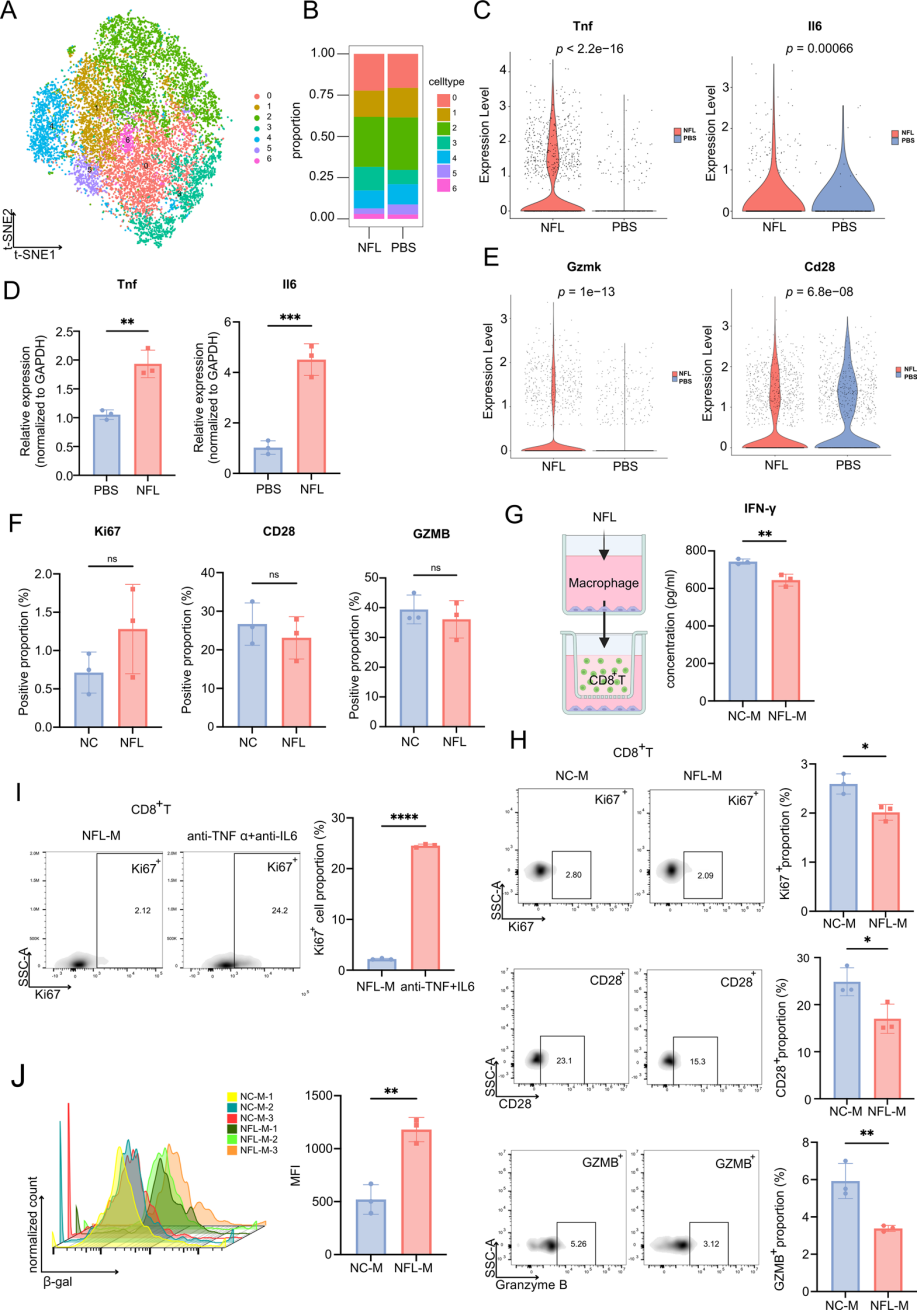
NFL-induced TAM enrichment promoted CD8^+^ T cell senescence. (**A**) t-SNE map showing macrophage populations following NFL or PBS treatment. (**B**) Proportional distribution of each macrophage cluster after treatment with NFL or PBS. (**C**) Expression of Tnf (left) and Il6 (right) in macrophages following NFL or PBS treatment. (**D**) Relative expression (normalized to GAPDH) of Tnf (left) and Il6 (right) in macrophages following NFL or PBS treatment, detected by RT-qPCR. (**E**) Expression of Gzmk (left) and Cd28 (right) in CD8^+^ T cells following NFL or PBS treatment. (**F**) Positive proportion of Ki67, CD28 and GZMB in CD8^+^ T cells following NFL or PBS treatment, detected by flow cytometry. (**G**) Schematic of non-contact co-culture between macrophages and CD8^+^ T cells, and proportion of GZMB^+^ CD8^+^ T cells in co-culture with macrophage-conditioned media after NFL or PBS treatment (left). The IFN-γ levels in the supernatant of CD8^+^ T cells, which were quantified by ELISA (right). (**H**)Representative flow cytometry plot (left) showing positive proportion of Ki67, CD28, GZMB in live CD8^+^ T cells treated with PBS or NFL. The right panel shows the proportion of positive cells within CD8^+^ live cells. (**I**) Representative flow cytometry plot (left) showing positive proportion of Ki67 in CD8^+^ T cells treated with macrophages’ supernatant with NFL treatment or additionally added neutralizing antibodies against TNF-α and IL-6. The right panel shows the proportion of Ki67^+^ cells within CD8^+^ live cells. (**J**) Flow cytometry histogram illustrating the expression of the senescence marker β-galactosidase (β-gal) in CD8^+^ T cells (left). Bar graph comparing the mean fluorescence intensity (MFI) of CD8^+^ T cell senescence marker induced by PBS-and NFL-treated macrophages (right). All data are presented as mean ± SD. Statistical significance was determined using a t-test. *p < 0.05, **p < 0.01, ***p < 0.001, ****p < 0.0001

To further investigate whether NFL-treated macrophages could influence CD8^+^ T-cell function, we extracted macrophages from the samples with the cell types annotated in Fig. 4E for additional analysis. Macrophages were clustered into seven distinct groups, with cluster 3 being significantly enriched in the NFL-treated group (Fig. 6A-B). This cluster exhibited higher expression levels of inflammatory cytokines, including Tnf, the gene name of TNF-α and Il6 (Fig. 6C and Figure S7A-B). To determine whether NFL would promote the upregulation of Tnf and Il6 expression in macrophages, we treated RAW264.7 cells with NFL and performed RT-qPCR analysis. The results showed that NFL significantly enhanced the expression of Tnf and Il6 in RAW264.7 cells (Fig. 6D).

Furthermore, differential analysis of CD8^+^ T-cells revealed elevated GZMK along with reduced CD28 expression both indicated T cell terminal differentiation and dysfunction in the NFL-treated group (Fig. 6E). We found that CD8^+^ T cells exhibited reduced proliferative capacity and weakened anti-tumor function in both the nerve lysate and NFL-treated groups, which is reminiscent of the characteristics of T cell senescence. Moreover, in the nerve lysate-treated model, similar reductions in CD28 expression were observed in CD8^+^ T cells (Figure S7C), further supporting the notion of T cell dysfunction and senescence.

In vitro, When we directly treated CD8^+^ T cells with NFL, we did not observe any significant changes in Ki67, CD28 and GZMB (Fig. 6F). However, when we treated macrophages from mouse spleen (macrophages were positively selected using CD11b magnetic beads) with NFL, both direct and indirect co-culture systems which performed with transwell insert yielded similar outcomes (Fig. 6G-H and S7D-H), indicating that NFL-stimulated MDM effects on CD8^+^ T cells were primarily mediated by soluble factors. To further investigate the effects of NFL-treated macrophages on CD8^+^ T cells, we collected the supernatant from macrophages treated with NFL and added neutralizing antibodies against TNF-α and IL-6. These antibodies were then applied to CD8^+^ T cells. Using flow cytometry (FC) to detect the expression of Ki67, we found that the Ki67 levels in CD8^+^ T cells were restored when neutralizing antibodies were used (Fig. 6I). This suggests that targeting TNF-α and IL-6 may help restore the proliferative capacity of CD8^+^ T cells.

**Fig. 7:**
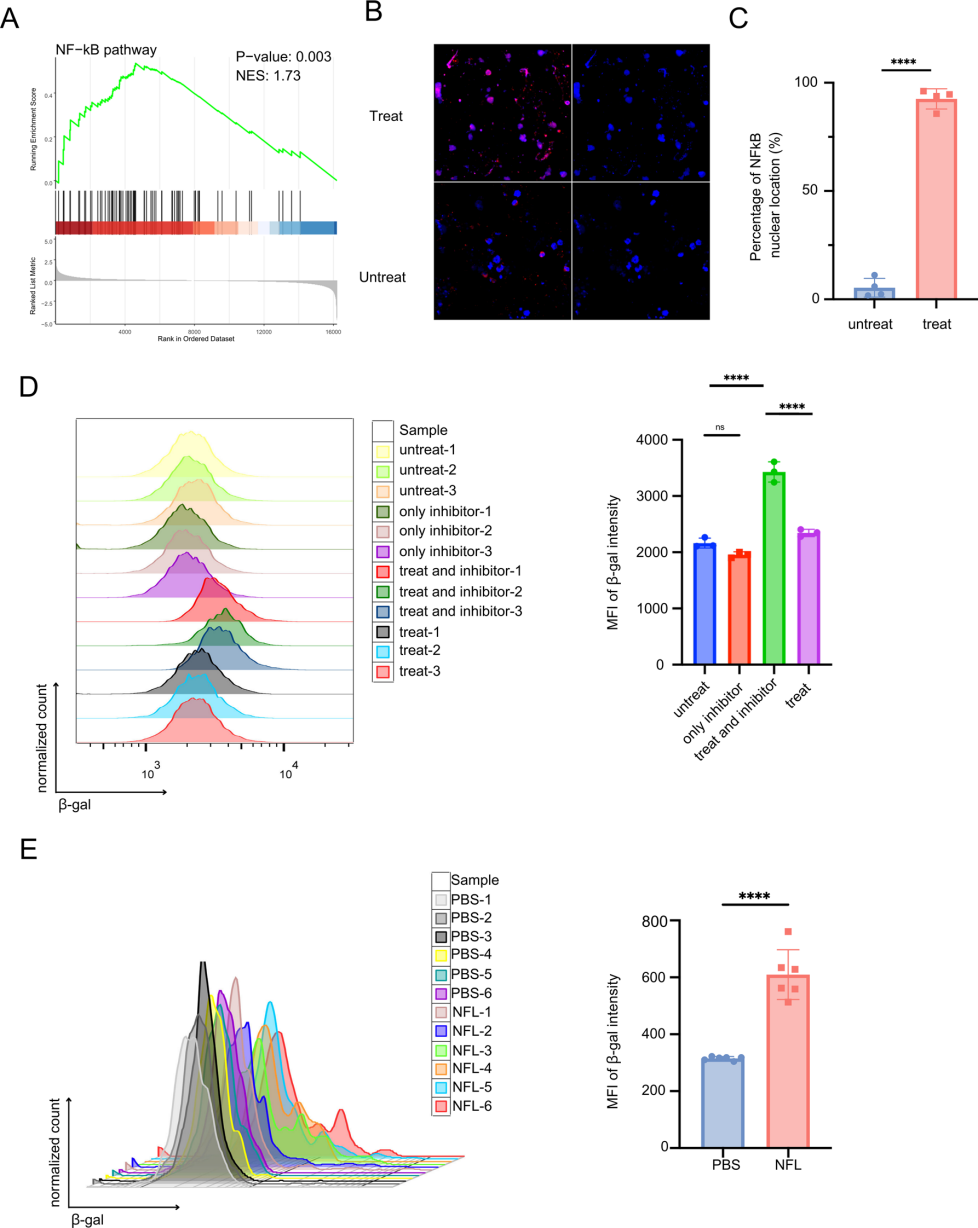
The supernatant of macrophages treated with NFL activates the NF-κB pathway in T cells, promoting cellular senescence. (**A**) GSEA enrichment analysis reveals that supernatants from macrophages treated with NFL activate the NF-κB pathway in CD8 ^+ ^T cells. (**B**) Immunofluorescence representative images shows the nuclear translocation of NF-κB in CD8^+^ T cells treated with supernatants from macrophages treated or untreated with NFL. (**C**) Quantitative analysis of (**B**) showing the percentage of CD8^ +^ T cells with NF-κB nuclear translocation (%) in the NFL-treated macrophage supernatant group versus untreated controls (*n* = 3 per group). (**D**) Flow cytometry histogram illustrating the expression of the senescence marker β-galactosidase (β-gal) in CD8^ +^ T cells treated with: supernatants from NFL-treated macrophages, supernatants from untreated macrophages, NF-κB inhibitor alone, or NFL-treated macrophage supernatants combined with NF-κB inhibitor. Right panel compares the mean fluorescence intensity (MFI) of β-gal in CD8 ^+^ T cells across these treatment groups (*n* = 3 per group). Data are presented as mean ± SD; statistical significance was determined by one-way ANOVA. (**E**) Flow cytometry histogram illustrating the expression of the senescence marker β-galactosidase (β-gal) in CD8^+^ T cells isolated from tumor tissues of mice treated with PBS or NFL (left), right panel shows the mean fluorescence intensity (MFI) of CD8^+^ T cell senescence marker induced by PBS-and NFL-treatment. All data are presented as mean ± SD. Statistical significance was determined using Student’s t-test for all panels except (**D**), where one-way ANOVA was applied (*p* < 0.05, *p < 0.01, **p < 0.001, ***p < 0.0001)

More interestingly, we used a Senescence-Tracker to determine whether the dysfunction of CD8^+^ T cells was related to their senescence. Flow cytometry results showed that the supernatant from NFL-treated macrophages indeed promoted the senescence of CD8^+^ T cells (Fig. 6J), while NFL alone failed to induce CD8^+^ T cell senescence (Figure S7I-J). The transcriptomic analysis comparing CD8^+^ T cells treated withNFL-activated versus control macrophage supernatant revealed significant NF-κB pathway activation (Fig. 7A-C and Figure S7K), establishing its central role in mediating CD8^+^ T cell dysfunction. NF-κB inhibition significantly reversed the suppressive effects of NFL-activated macrophage supernatant on CD8^+^ T cell function (Fig. 7D). Additionally, when we performed flow cytometry to evaluate the senescence phenotype of CD8^+^ T cells in the NFL-treated mouse model, the results revealed a more pronounced senescence in the NFL-treated group (Fig. 7E). This finding highlights the potential role of macrophage-derived cytokines in modulating CD8^+^ T cell function and senescence.

**Fig. 8:**
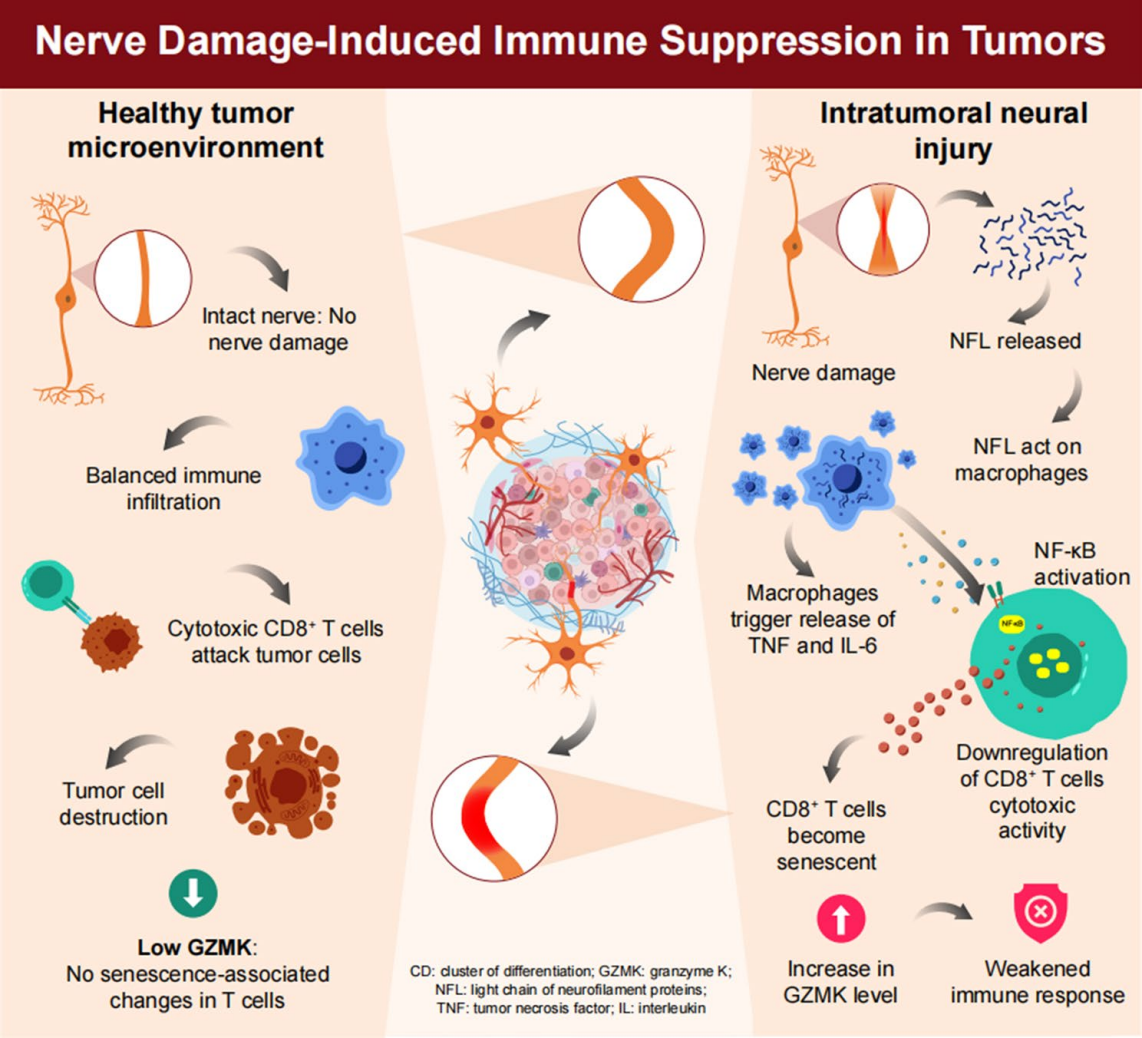
Schematic diagram of our main findings. The illustration of the breast cancer immune microenvironment with or without peripheral nerve injury

These changes in both NFL-treated macrophages and CD8^+^ T cells suggest that NFL-induced TAM enrichment may accelerate CD8^+^ T cell senescence by releasing inflammatory cytokines that impair T-cell function and contribute to immune evasion in the tumor microenvironment (Fig. 8).

## Discussion

Peripheral nerve regeneration, primarily regulated by Schwann cells, is a well-established model for studying nerve repair [34]. While prior research has focused on nerve regeneration in conditions like diabetes [35], epilepsy, and chronic pain [36], the role of nerve damage and repair within the tumor microenvironment (TME) remains poorly understood. Our study reveals that nerve injury in tumors triggers TME alterations that drive tumor growth and immune evasion. Unlike tightly regulated physiological nerve regeneration, breast cancer-associated nerve damage leads to the release of neurofilament light chain (NFL), creating a unique immunosuppressive niche that accelerates tumor progression. GZMK overexpression has been linked to T-cell terminal differentiation and dysfunction [37, 38]. T cell senescence refers to the loss of normal proliferative capacity, reduced antigen reactivity, and increased secretion of inflammatory cytokines, all of which impair the ability of T cells to recognize and kill tumor cells, ultimately inhibiting antitumor immunity [39, 40]. CD28 is an important co-stimulatory molecule on the surface of T cells, and its decreased expression is a hallmark of immune senescence [41]. The supernatant from NFL-treated macrophages significantly modulated the expression of GZMK and CD28 in CD8^+^ T cells, suggesting that NFL plays a critical role in shaping the immunosuppressive microenvironment that drives T cell dysfunction. This suggests that dynamic neural regulation in the TME may form a novel tumor-promoting network independent of classical inflammatory pathways.

A limitation of our study is the use of the breast cancer cell line, which induces rapid tumor progression after orthotopic injection and thus may not fully recapitulate the prolonged nerve regeneration process observed in human tumors. Future work should employ long-term tumor models to better simulate tumor-induced nerve injury, neurogenesis, and nerve interactions within the TME.

During tissue repair, macrophage proliferation and migration are typically coupled. Consistent with Wallerian degeneration, nerve lysate treatment enhanced both processes, suggesting that nerve injury promotes macrophage-mediated tissue repair mechanisms. The infiltration of tumor-associated macrophages increases following nerve injury, a phenomenon that is likely driven by the complex roles that macrophages play in nerve injury and repair [42, 43]. Macrophages are involved in clearing axonal debris and disintegrating myelin sheaths, creating a favorable environment for nerve regeneration [44]. They also secrete cytokines and growth factors that regulate angiogenesis and Schwann cell activity, which are crucial for the repair process [42]. Upon nerve injury, macrophages adopt a proliferative phenotype that supports repair mechanisms [45, 46]. Intriguingly, increased infiltration of tumor-associated macrophages (TAMs) following nerve injury implies that nerve damage may foster an immunosuppressive environment conducive to tumor progression.

Our findings redefine NFL—a biomarker of neuronal injury—as an active contributor to tumor immune editing. In breast cancer, NFL enhances TAM infiltration and induces CD8^+^ T cell senescence, facilitating immune evasion. This challenges the conventional view of NFL as a passive injury marker and reveals its direct immunomodulatory role. Notably, unlike tumor-associated neurons that modulate immunity via classical neurotransmitters [5], the immunosuppressive effects of NFL are neurotransmitter receptor-independent, suggesting the existence of previously unrecognized neuro-immune communication pathways in tumors. Solid tumors are complex ecosystems where heterogeneity demands holistic analysis of all components [47, 48]. Despite their emerging significance, tumor-associated nerve fibers in non-CNS malignancies remain understudied. Our work highlights how nerve damage regulates immune evasion in breast cancer, deepening understanding of the tumor ecosystem. Crucially, protecting tumor-infiltrating nerves from injury may reverse immune suppression, offering a novel strategy to inhibit progression. These findings advocate for therapeutic interventions targeting nerve damage in tumors. We will further explore NFL’s role across tumor types and develop NFL-targeted therapies to disrupt its tumor-promoting effects.

## Electronic supplementary material

Below is the link to the electronic supplementary material.

Supplementary Material 1

## Data Availability

The scRNA-seq raw data have been deposited in the NCBI BioSample database with the accession numbers SAMN49890210, SAMN49890211, SAMN49890212, SAMN49890213, SAMN49890214, and SAMN49890215. Additionally, the RNA-seq data have been submitted to the Gene Expression Omnibus (GEO) and are accessible through the GEO Series accession number GSE306312.

## Abbreviations

NFL: Neurofilament light chain
TAM: Tumor-associated macrophage
PNS: Peripheral nervous system
TCGA-BRCA: The cancer genome atlas breast cancer dataset
TCGA: The cancer genome atlas
OS: Overall survival
TME: Tumor microenvironment
DC: Dendritic cell
6-OHDA: 6-hydroxydopamine
mIHC: Multiplex immunohistochemistry
scRNA-seq: Single-cell RNA sequencing
PBMC: Peripheral blood mononuclear cells
BMDM: Bone marrow-derived macrophages
MDM: Monocyte-derived macrophages
IFN-γ: Interferon-gamma
TNF-α: Tumor necrosis factor-alpha
