# Supplementary material for: Peripheral nerve injury-induced remodeling of the tumor-associated macrophages promotes immune evasion in breast cancer
Source: J Exp Clin Cancer Res. 2025 Oct 6;44:280. doi: 10.1186/s13046-025-03545-x (PMC12502561; doi:10.1186/s13046-025-03545-x)
Supplement: Supplementary file 1 — Supplementary Material 1 [file 13046_2025_3545_MOESM1_ESM.docx]

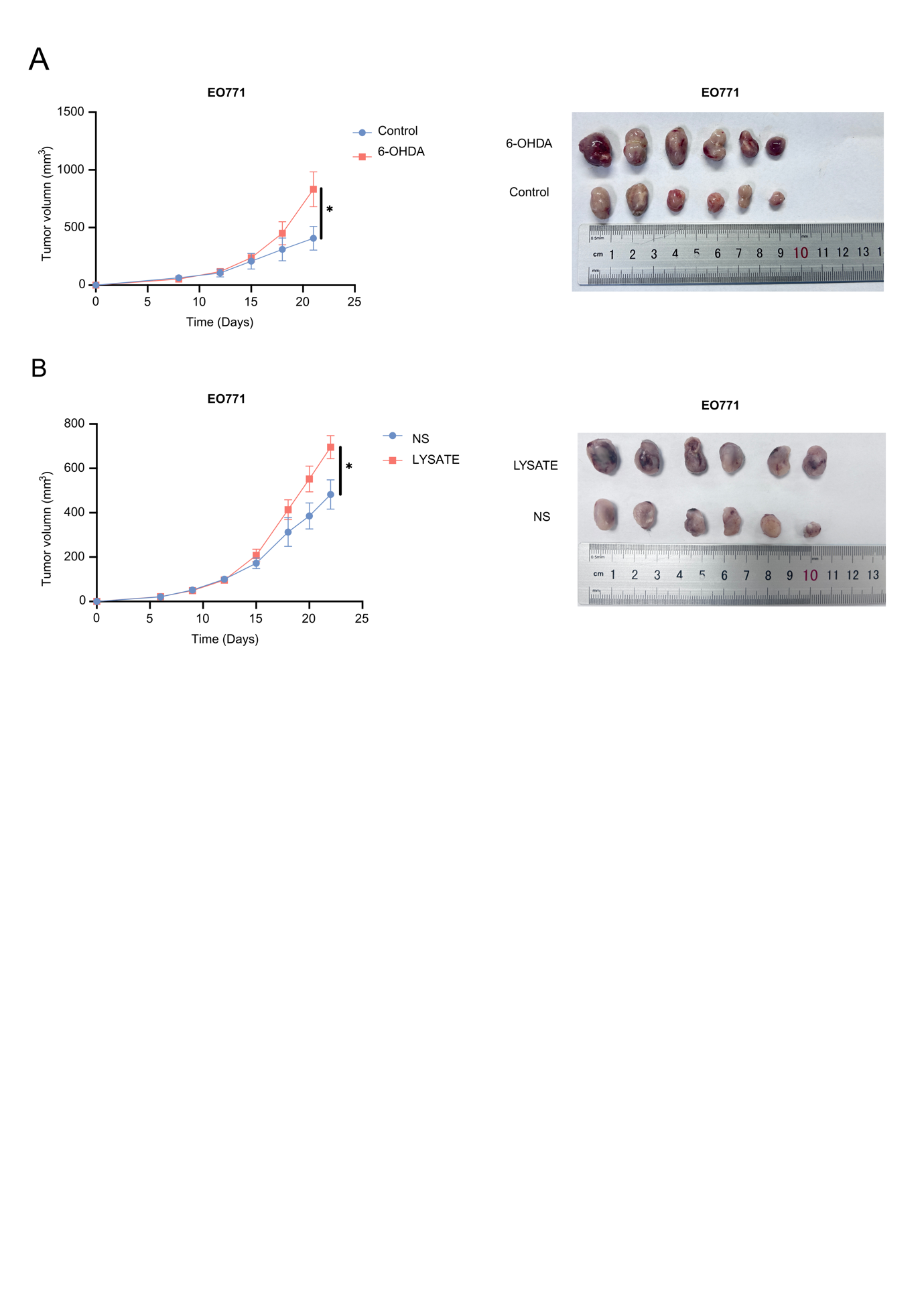


**Fig.S1**

(A) Tumor growth curve of EO771 tumor in C57BL/6 mice treated with 6-OHDA or normal saline (left). Images of excised tumors on the final day with individual tumor sizes shown (right). (*n*=6 each group.)

(B) Tumor growth curve of EO771 tumor in C57BL/6 mice treated with nerve lysate or normal saline (left). Images of excised tumors on the final day with individual tumor sizes shown (right). (*n*=6 each group.)

Data is presented as mean ± SEM. Statistical significance was determined by two-tailed student’s t-test. **p* < 0.05, ***p* < 0.01, ****p* < 0.001, *****p* < 0.0001.


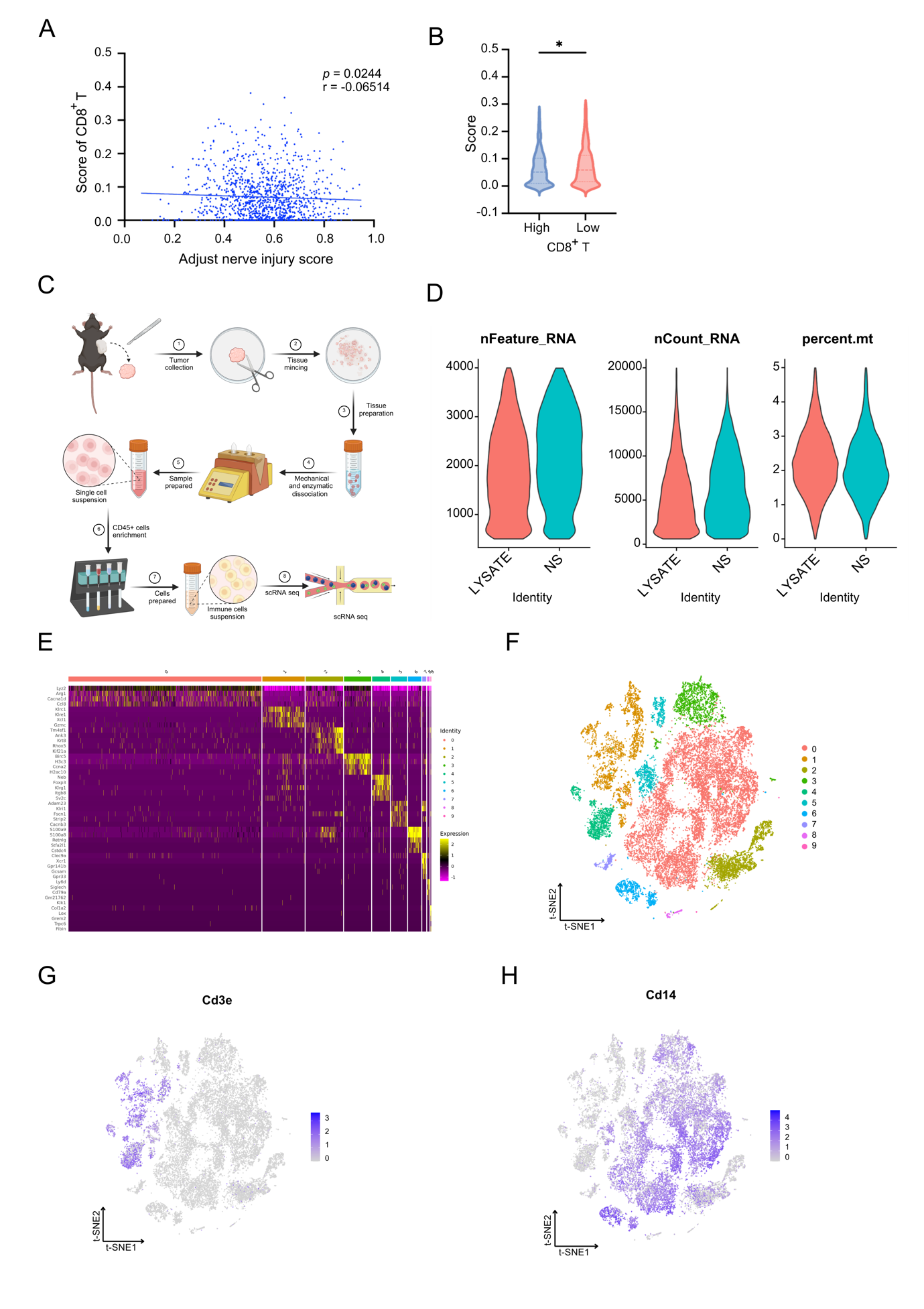


Fig.S2 **Peripheral Neural Injury Is Associated with Poor Prognosis and Promotes Breast Cancer Growth**

(A) Correlation between the infiltrating score of CD8^+^ T cells and the degree of nerve injury in TCGA-BRCA patient samples. The correlation coefficient (r) and p-value are indicated.

(B) CD8+ T cell infiltration scores in TCGA-BRCA patients, stratified by high or low degrees of nerve injury (*p < 0.05).

(C) Workflow for isolating immune cells from mouse tumors and subsequent scRNA-seq analysis.

(D) Cell Quality Filtering result for scRNA-seq, including the number of genes detected (nFeature_RNA), the total count of RNA molecules (nCount_RNA), and the percentage of mitochondrial genes (percent.mt).

(E) Heatmap showing the top differentially expressed genes in immune cells.

(F) t-SNE map of immune cells populations.

(G) Feature plot showing the expression of *Cd3e* in immune cells from tumor tissues.

(H) Feature plot showing the expression of *Cd14* in immune cells from tumor tissues.

Statistical significance was determined using unpaired two-tailed t-test. **p* < 0.05.


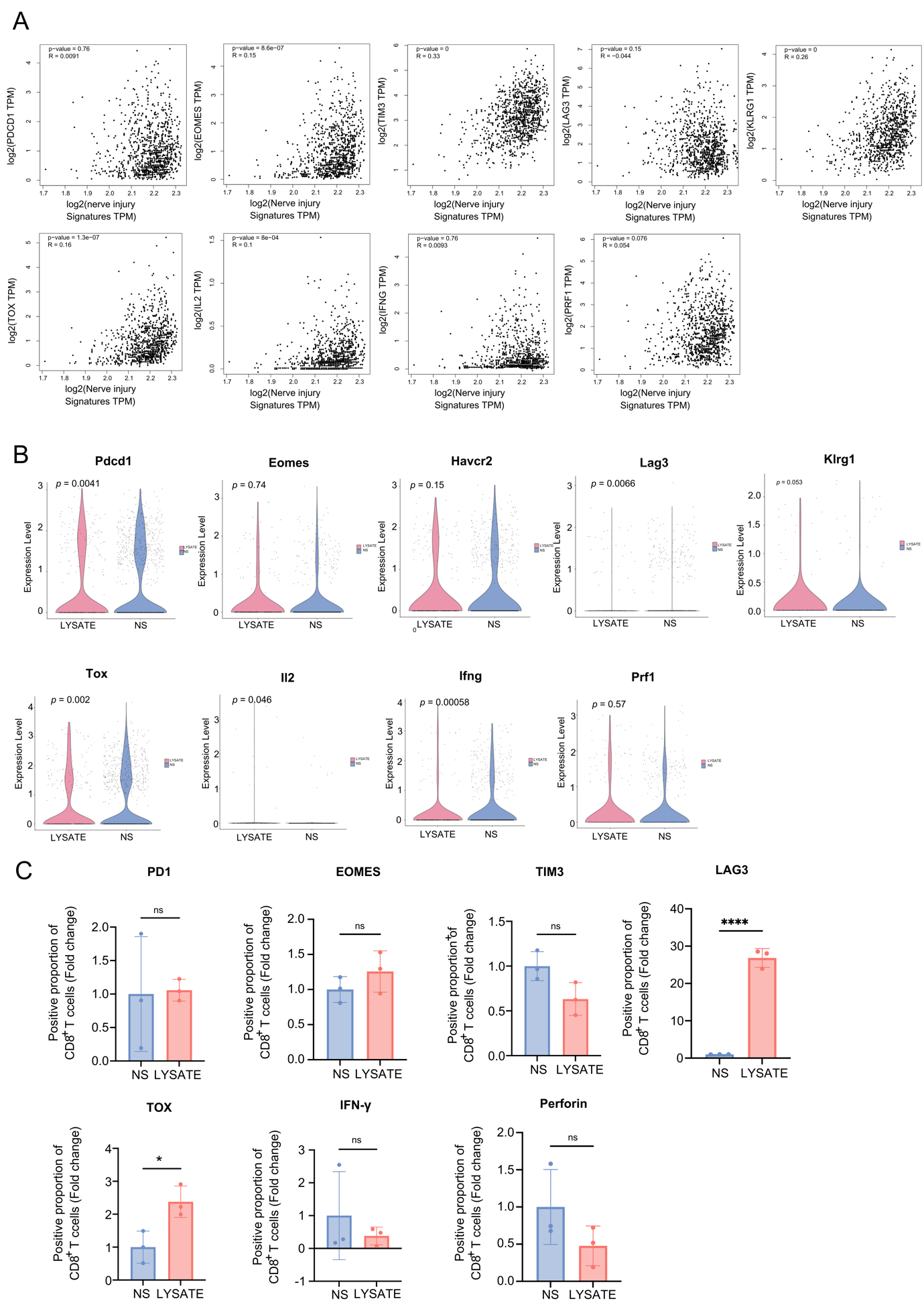


Fig.S3 **Correlation of Nerve Injury with CD8**^+^ **T Cell Exhaustion and Functional Markers**

(A) Correlation between mRNA levels of CD8^+^ T cell exhaustion-associated molecules (PD1, EOMES, TIM3, LAG3, KLRG1, TOX) and cytokines (IL-2, IFNG, PRF1) with nerve injury scores in TCGA-BRCA patient samples. Correlation coefficients (R) and p-values indicate the significance and strength of the associations.

(B) mRNA expression levels of CD8^+^ T cell exhaustion-associated molecules (PD1, EOMES, TIM3, LAG3, KLRG1, TOX) and cytokines (IL-2, IFNG, PRF1) in tumor-infiltrating CD8+ T cells of normal saline- or nerve lysate-treated group, determined by scRNA-seq.

(C) Fold change in the proportion of CD8⁺ T cells expressing PD-1, Eomes, Tim-3, Lag-3, Tox, IFN-γ, and perforin in nerve-lysate-treated tumors relative to normal-saline controls, determined by immunofluorescence. (n=3 each group)

Statistical significance was determined using unpaired two-tailed t-test. ns means no significance, **p* < 0.05.


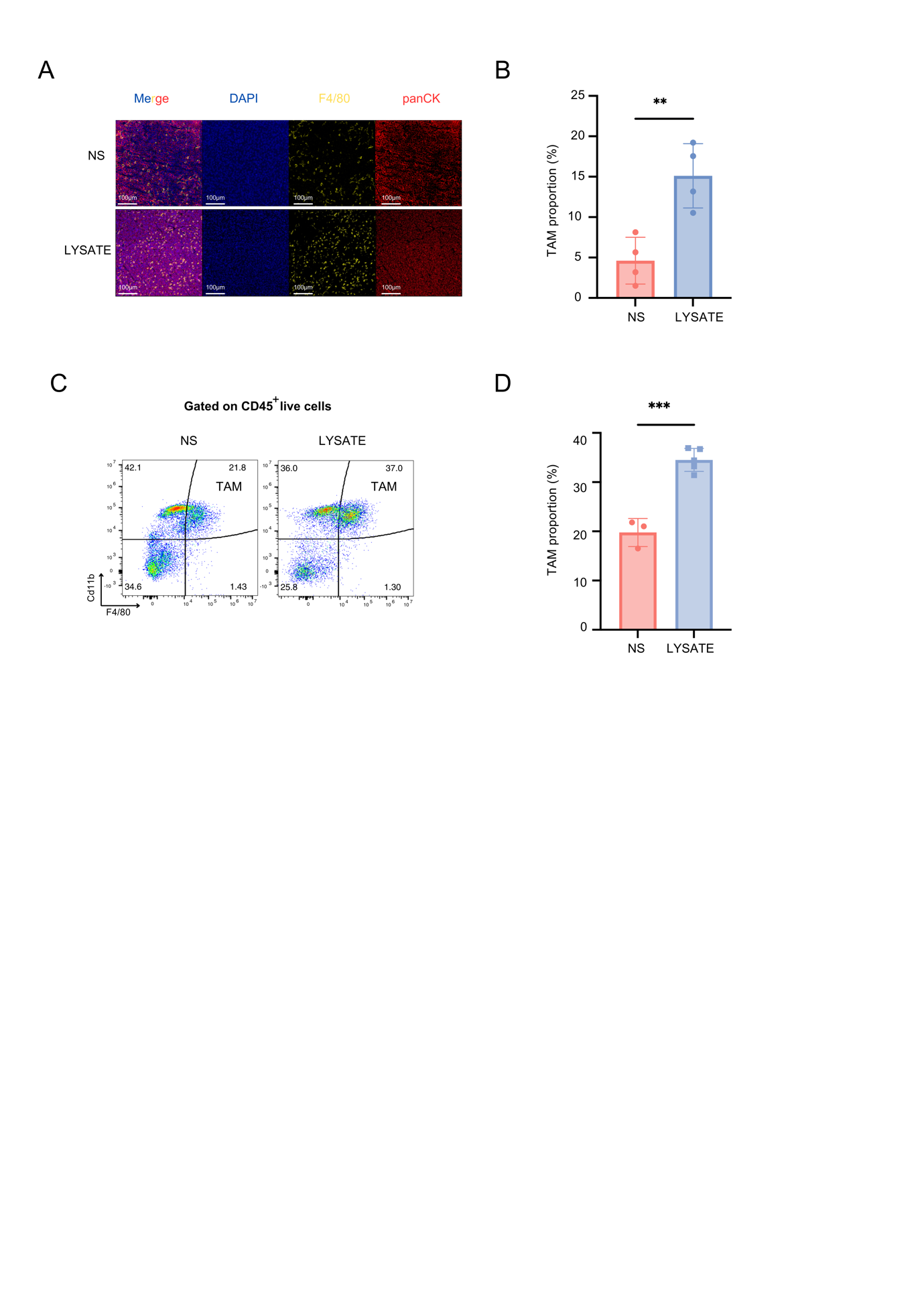
Fig.S4 **Nerve lysate treatment increases tumor-associated macrophages (TAMs)**

(A) Representative immunofluorescent images of F4/80 and panCK staining of tumor tissue treated with NS or nerve lysate.

(B) Quantification of the proportion of TAMs within total cells in tumor sections treated with NS or nerve lysate. *n*_NS_=4, *n*_LYSATE_=4.

(C) Representative flow cytometry plot showing TAM (CD11b^+^F4/80^+^) cells from tumor tissues treated with NS or nerve lysate.

(D) Proportion of TAM (CD11b^+^F4/80^+^) cells within total live CD45^+^ cells in tumor tissues from tumor tissue treated with NS or nerve lysate. *n*_NS_=3, *n*_LYSATE_=5.

All data are presented as mean ± SD. Statistical significance was determined using two-tailed unpaired Student’s t-test. ***p* < 0.01, ****p* < 0.001.


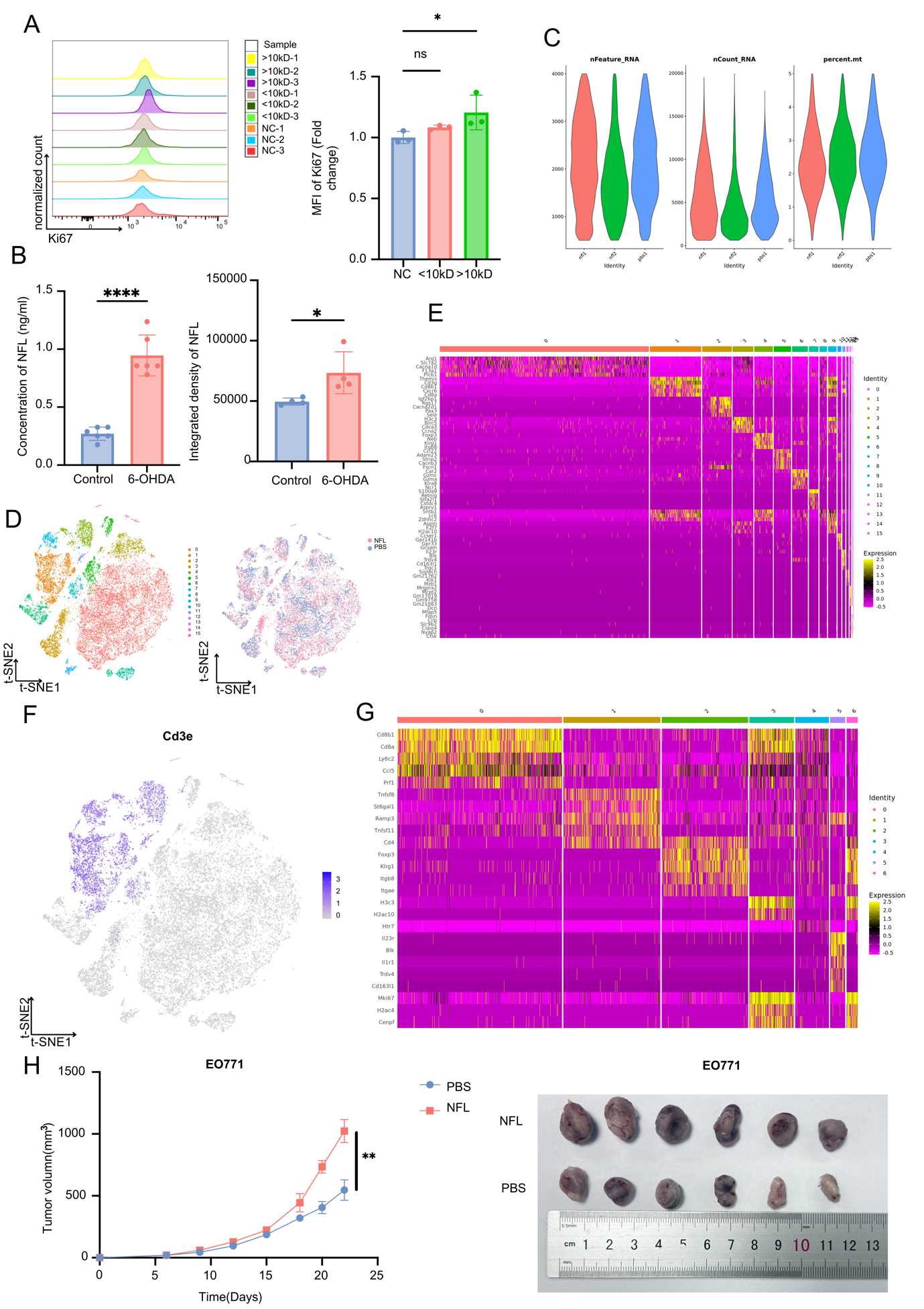
Fig.S5 **Immune profiling of tumor-infiltrating cells after NFL or PBS treatment**

(A) Flow cytometry histogram showing Ki67 expression in macrophages (left). Bar graph comparing normalized mean fluorescence intensity (MFI) of Ki67 in macrophages induced by PBS (NC), >10kD fraction and <10kD fraction (right). N=3 per group. Statistical comparisons were performed by one-way ANOVA. ns = not significant, **p* < 0.05.

(B) Concentration of NFL in tumor homogenates as determined by ELISA (left). (*n*=6 per group). Quantification of neurofilament light (NFL) immunoreactivity in tumor tissue. NFL integrated density was measured on IHC-stained sections using the Color Deconvolution plugin in Fiji software; values are shown for mice treated with intraperitoneal 6-OHDA versus normal saline (Control). *n*=4 each group (right). Values are presented as mean ± SD. Statistical significance was assessed by unpaired two-tailed t-test (**p* < 0.05)

(C) Cell Quality Filtering result for scRNA seq, including nFeature_RNA, nCount_RNA and percent_mt.

(D) t-SNE map of clustered immune-cell populations from tumor tissues. Lower inset shows sample origin (PBS or NFL) overlaid on the same t-SNE to display sample distribution.

(E) Heatmap of the top differentially expressed genes across immune-cell clusters.

(F) Feature plot showing Cd3e expression in immune cells across the t-SNE map.

(G) Heatmap of the top differentially expressed genes within T cell clusters.

(H) Tumor growth curve of EO771 tumor in C57BL/6 mice treated with NFL or PBS (left). Images of excised tumors on the final day with individual tumor sizes shown (right). *n*=6 each group.

Data are presented as mean ± SD. Statistical comparison between groups at the final time point was performed using two‑tailed unpaired Student’s t‑test. **p* < 0.05, **p < 0.01.


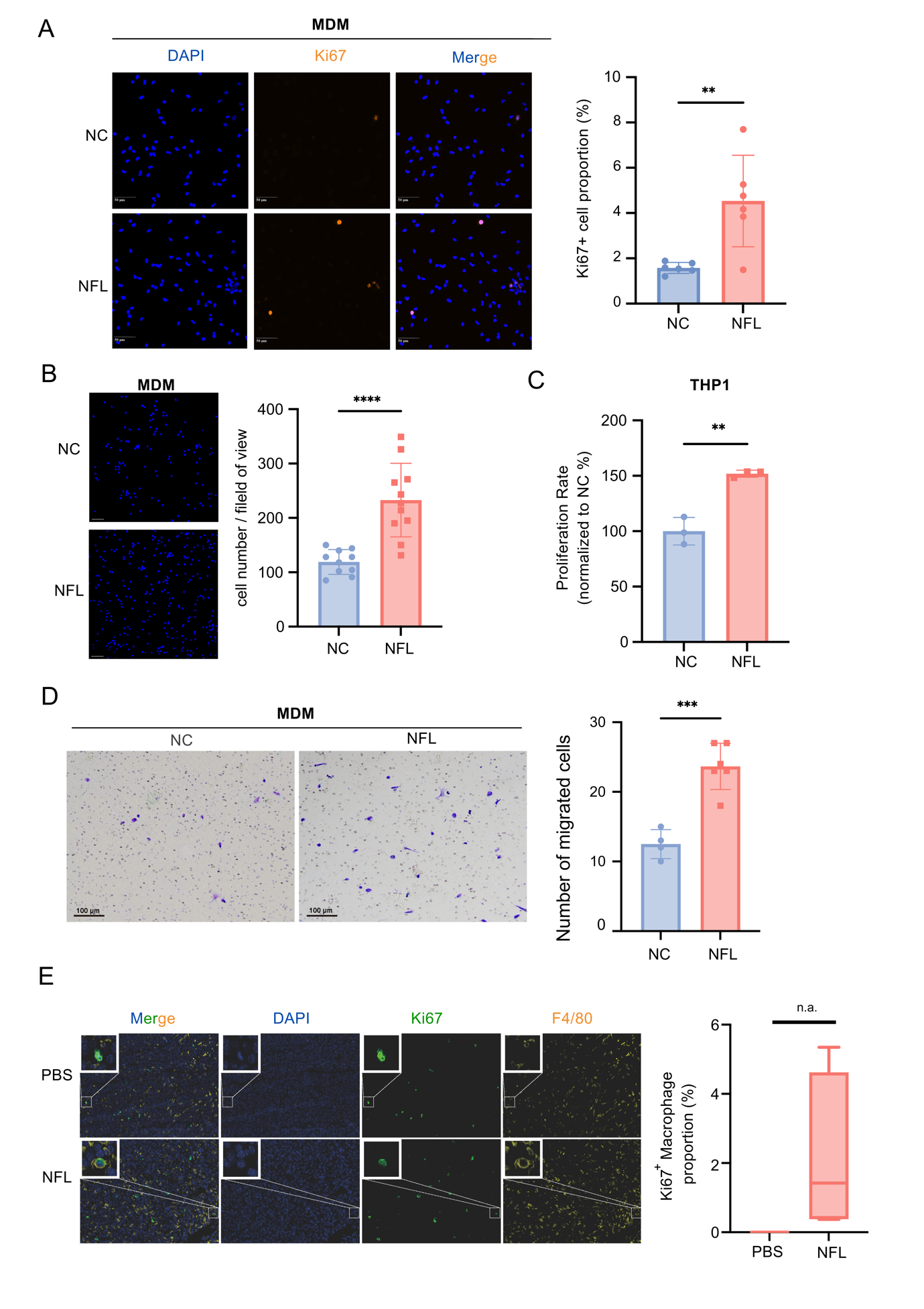


Fig.S6 **NFL induces proliferation and migration of monocyte‑derived macrophages (MDMs)**

(A) Representative immunofluorescent images of Ki67 and DAPI staining in monocyte derived macrophages (MDMs) treated with PBS or NFL. The right panel shows the proportion of Ki67^+^ MDMs, *n*=6 each group.

(B) Representative DAPI images of MDM cultures after treatment (left). Cell count per fileld of view after treatment (right), *n*_NC_=10, *n*_NFL_=11.

(C) Proliferation rate of THP‑1 cells treated with NFL or PBS (NC) measured by CCK‑8 assay, *n*=3 each group.

(D) Representative images from transwell migration assays of MDMs stained by crystal violet (left) and quantification of migrated cells per field (right) of MDMs treated with NFL or PBS, *n*_NC_=4, *n*_NFL_=5.

(E) Representative immunofluorescent images of Ki67 and F4/80 staining (left) and the in proportion of Ki67^+^F4/80^+^ cells among all macrophages in tumor tissues from mice treated with PBS or NFL, n = 3 each group. All values in the PBS group were zero, therefore an unpaired two‑tailed t‑test is not applicable (n.a.) and no p‑value is reported.

Data in (E) is presented as median (min–max), all other data are presented as mean ± SD. Statistical significance was performed using two‑tailed unpaired Student’s t‑test. **p < 0.01, ***p < 0.001, ****p < 0.0001.


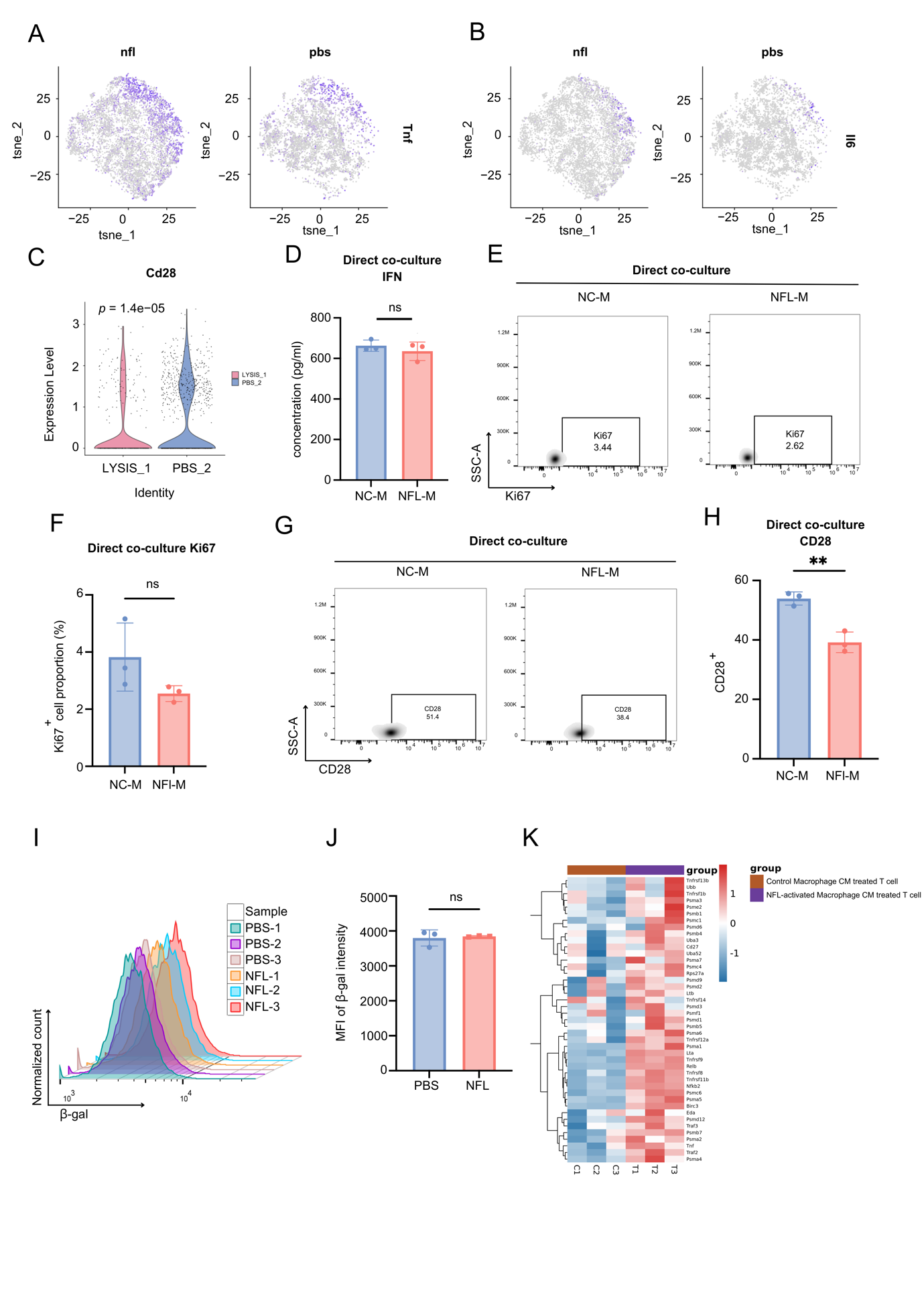


Fig.S7 **NFL-induced TAM enrichment promoted CD8^+^ T cell senescence.**

(A) Feature plot showing the expression of *Tnf* in macrophage clusters after treatment with NFL or PBS.

(B) Feature plot showing the expression of *Il6* in macrophage clusters after treatment with NFL or PBS.

(C) Expression of *Cd28* in macrophages following NS or nerve lysate treatment.

(D) Splenic macrophages from mice treated with or without NFL were co‑cultured in direct contact with CD8+ T cells. After co‑culture, supernatants from the CD8+ T cell fraction were collected and IFN‑γ concentration was measured by ELISA.

(E) Representative flow cytometry plot showing positive proportion of Ki67 staining in live CD8^+^ T cells after direct contact co‑culture with macrophages (left: control; right: NFL‑treated macrophages).

(F) Quantification of the proportion of Ki67^+^ cells among live CD8+ T cells from (E).

(G) Representative flow cytometry plot showing positive proportion of CD28 staining in live CD8^+^ T cells after direct contact co‑culture with macrophages (left: control; right: NFL‑treated macrophages).

(H) Quantification of the proportion of CD28^+^ cells among live CD8+ T cells from (G).

(I) Flow cytometry histogram overlays illustrating the expression of the senescence marker β-galactosidase (β-gal) in CD8^+^ T cells treated with PBS and NFL directly.

(J) Mean fluorescence intensity (MFI) of β‑gal staining in CD8^+^ T cells from (I).

(K) Heatmap showing transcriptional changes in CD8^+^ T cells following treatment with conditioned media from NFL‑activated macrophages versus control macrophages.

All data are presented as mean ± SD, *n* = 3. Statistical comparison was performed using two‑tailed unpaired Student’s t‑test. ns = not significant, **p < 0.01.
